# Comparing the gut microbiome along the gastrointestinal tract of three sympatric species of wild rodents

**DOI:** 10.1038/s41598-021-99379-6

**Published:** 2021-10-07

**Authors:** Jason L. Anders, Mohamed Abdallah Mohamed Moustafa, Wessam Mohamed Ahmed Mohamed, Takashi Hayakawa, Ryo Nakao, Itsuro Koizumi

**Affiliations:** 1 Graduate School of Environmental Science, Hokkaido University, N10W5, Sapporo, Hokkaido 060-0810 Japan; 2 Laboratory of Parasitology, Graduate School of Infectious Diseases, Faculty of Veterinary Medicine, Hokkaido University, Kita-18, Nishi-9, Sapporo, Hokkaido 060-0818 Japan; 3 Department Animal Medicine, South Valley University, Qena, 83523 Egypt; 4 Faculty of Environmental Earth Science, Hokkaido University, N10W5, Sapporo, Hokkaido 060-0810 Japan; 5 Japan Monkey Centre, Inuyama, Aichi 484-0081 Japan

**Keywords:** Community ecology, Microbial ecology

## Abstract

Host–microbe interactions within the gastrointestinal tract (GIT) play a pivotal role in shaping host physiology, ecology, and life history. However, these interactions vary across gut regions due to changes in the physical environment or host immune system activity, thereby altering the microbial community. Each animal species may harbor their own unique microbial community due to host species-specific ecological traits such as dietary habits, micro-habitat preferences, and mating behavior as well as physiological traits. While the gut microbiota in wild animals has received much attention over the last decade, most studies comparing closely related species only utilized fecal or colon samples. In this study, we first compared the gut microbial community from the small intestine, cecum, colon, and rectum within three sympatric species of wild rodents (i.e. *Apodemus speciosus*, *A. argenteus*, and *Myodes rufocanus*). We then compared each gut region among host species to determine the effect of both gut region and host species on the gut microbiota. We found that the small intestine harbored a unique microbiome as compared to the lower GIT in all three host species, with the genus *Lactobacillus* in particular having higher abundance in the small intestine of all three host species. There were clear interspecific differences in the microbiome within all gut regions, although some similarity in alpha diversity and community structure within the small intestine was found. Finally, fecal samples may be appropriate for studying the lower GIT in these species, but not the small intestine.

## Introduction

The vertebrate gastrointestinal tract (GIT) is a complex ecosystem occupied by a diverse community of bacteria that impact many aspects of the host’s biology such as behavior^1^, digestion^2^, and immune system function through interactions with the host^3–5^. Therefore, understanding host-microbe interactions will help us to better understand the ecology and evolution of wildlife^6^. However, interactions are not uni-directional, as the host helps shape the microbial community by actively destroying species that are pathogenic while allowing those that are beneficial to remain or tolerating those that cause no harm^7^. Due to species-specific physiological and dietary needs, this selective process leads to unique microbial community profiles even among sympatric species exposed to the same environmental bacteria^8^ and often mirroring their evolution^9–11^. Although this phylosymbiosis has already been demonstrated in several groups of taxa, only two studies have done so using multiple gut regions from the same individuals in lizards^12^ and rodents (primarily mice)^10^, with the later using laboratory reared animals.

The digestive tract is a complex environment that changes drastically in physical structure, immune system activity, oxygen concentration, and pH going from the oral cavity to the anus due to differing physiological functions as required by the host^13^. This creates physical and physiological barriers that bacteria must cross before establishing themselves. Therefore, unique microbial communities reside within each gut region, with those in the upper and lower digestive tract being distinctly different from each other as has been demonstrated in several groups of animals such as rodents^10,14,15^, pigs^16^, chickens^17^, and lizards^12^. Although we are only just beginning to understand the biogeography of the gut microbiota along the digestive tract of vertebrates, this has called into question the wide spread use of fecal samples to answer all manner of questions regarding the gut microbiome^11,15,17^. Fecal samples are easy to collect non-invasively and may provide a representation of the gut microbial flora in the lower GIT, particularly species membership. However, it may not accurately reflect species abundances especially of the upper GIT^17^.

In this study, we investigated the gut microbial communities of three sympatric species of wild caught rodents, two field mice (*Apodemus speciosus* and *A. argenteus*) and one vole (*Myodes rufocanus*), to determine differences in the microbiome among four gut regions (i.e. small intestine, cecum, colon, and the rectum) within each host species, as well as among species differences within the same gut regions. Both *A. speciosus* and *A. argenteus* are common throughout the Japanese archipelago^18^. Although they maintain overlap in their ecological niches, *A. speciosus* is entirely ground dwelling (as is *M. rufocanus*) while *A. argenteus* is often arboreal, especially when the population density of *A. speciosus* is high^19^. *M. rufocanus* on the other hand, is widely distributed across Eurasia from Fennoscandia to Japan where it is only found within Hokkaido and associated small islands^20^. All three species are omnivorous, but the diet of *A. speciosus* and *A. argenteus* largely consists of nuts, seeds, and insects while that of *M. rufocanus* is dominated by herbaceous plants and bamboo^21,22^.

We hypothesized that within species, each gut region would harbor a unique microbiome, particularly between the small intestine and the lower GIT (i.e. cecum, colon, and rectum) because of differences in host physiological function^13,14^. Due to different life and evolutionary histories of each host species we predicted that all gut regions would show significant among species differences, with the largest between *M. rufocanus* and both species of field mice. Lastly, as our rectum samples were fecal matter taken directly from the GIT rather than after defecation, we wanted to see if the microbiome was an accurate representation of any specific gut region. We expected it to be most similar to the microbiome of the colon. By answering these questions, we hope to better understand the role that gut region and host species have in shaping the gut microbiome of wild animals.

## Results

### Host and gut content sampling

A total of 94 individuals (42 *A. speciosus*, 9 *A. argenteus*, and 43 *M. rufocanus*) were captured from four sites within the Kamikawa Chubu national forest in the central area on the island of Hokkaido, Japan (Supplementary Table S1), and a total of 280 gut content (from the small intestine, cecum, and colon) and fecal matter (from the rectum) samples were collected for microbiome analysis (Supplementary Table S2). Based on 16S rRNA amplicon sequencing using Illumina Miseq, a total of 12,286,171 paired-end reads were obtained after quality filtering and chimeric sequence removal. There was an average of 43,879 reads per sample, although it varied among species and gut region (Supplementary Table S3).

### Within host species/among gut region gut microbiota alpha diversity

**Figure 1:**
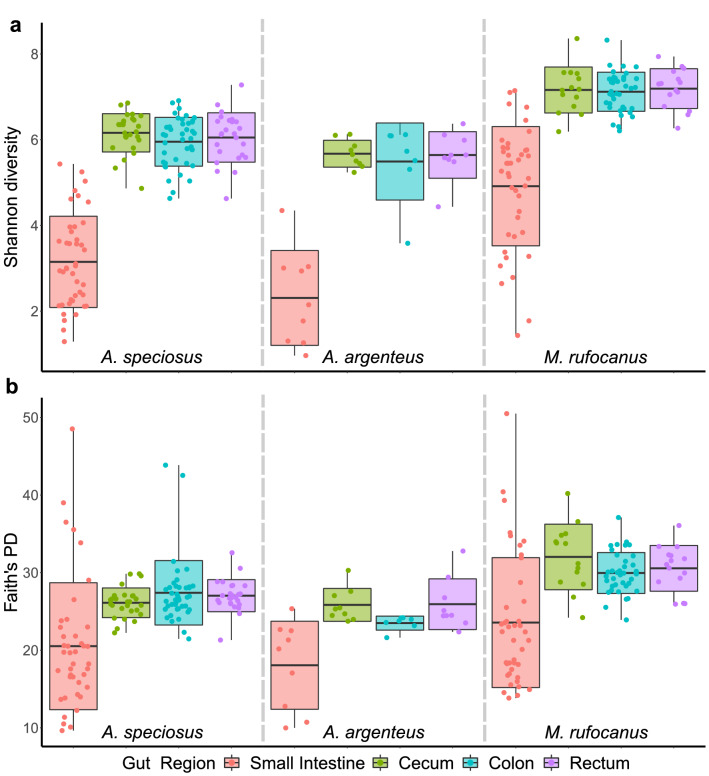
Alpha diversity within each gut region of each species based on (**a**) Shannon diversity and (**b**) Faith’s PD. Dashed lines separate host species.

Alpha diversity of the gut microbiota in the small intestine was significantly lower than the rectum, colon, and cecum in all three host species based on Shannon diversity, Faith’s PD, evenness, and number of ASVs as expected (GLME: all *p* < 0.01; Fig. 1, Supplementary Fig. S2, Supplementary Tables S4–S7). There was no difference in alpha diversity among the cecum, colon, or rectum within any species (GLME: all *p* > 0.05; Fig. 1, Supplementary Fig. S2, Supplementary Tables S4–S7). Males had significantly higher alpha diversity within all gut regions of *A. speciosus* while female *A. argenteus* had significantly higher alpha diversity as compared to males (GLME, all *p* < 0.02; Supplementary Tables S4–S7). There was no effect of sex on gut microbiota alpha diversity in any gut region of *M. rufocanus* (GLME: all *p* > 0.05; Supplementary Tables S4–S7) while age had no effect in any gut region of any rodent species (GLME: all *p* > 0.05; Supplementary Tables S4–S7).

### Among host species alpha diversity

*Myodes rufocanus* had significantly higher alpha diversity in all four gut regions as compared to both *A. speciosus* and *A. argenteus* based on all four diversity measurements (GLME: all *p* < 0.01; Fig. 1, Supplementary Fig. S2, Supplementary Tables S8–S11) except for Faith’s PD of the small intestine (GLME: *A.* speciosus: b = − 0.057, SE = 0.095, *p* = 0.55; *A. argenteus*: b = − 0.146, SE = 0.154, *p* = 0.346; Fig. 1, Supplementary Table S9). There were fewer significant differences in alpha diversity between *A. speciosus* and *A. argenteus* as expected with the colon exhibiting differences based on Faith’s PD and evenness, as well as in the small intestine and cecum for Shannon diversity and evenness (GLME: all *p* < 0.05; Fig. 1, Supplementary Fig. S2, Supplementary Tables S8–S11). There were no significant differences in alpha diversity within the rectum between *Apodemus* spp., nor was there an effect of age or sex on any alpha diversity measurement in any among species analysis (Fig. 1, Supplementary Fig. S2, Supplementary Tables S8–S11).

### Within host species/among gut region beta diversity

When testing for the effect of gut region on microbiome beta diversity within each species when all gut regions were included for PERMANOVA, we found gut region had a highly significant effect in all three host species regardless of distance metric (PERMANOVA: all *p* < 0.01; Supplementary Tables S12–S14). Field site also significantly impacted beta diversity in all three rodent species (PERMANOVA: all *p* < 0.03; Supplementary Tables S12–S14) though the effect size was smaller than it was for gut region except for *A. argenteus* according to both Jaccard and Bray–Curtis distance metrics (Supplementary Tables S12–S14). Age significantly impacted beta-diversity in both *A. speciosus* and *M. rufocanus* according to all four diversity metrics (PERMANOVA: all p < 0.01; Supplementary Tables S12, S14) while sex was significant for all except weighted UniFrac in all three species (PERMANOVA: *p* = 0.055 to 0.266; Supplementary Tables S12–S14) as well as unweighted UniFrac in *M. rufocanus* (PERMANOVA: R^2^ = 0.011 F = 1.6, *p* = 0.071; Supplementary Table S14).

**Figure 2:**
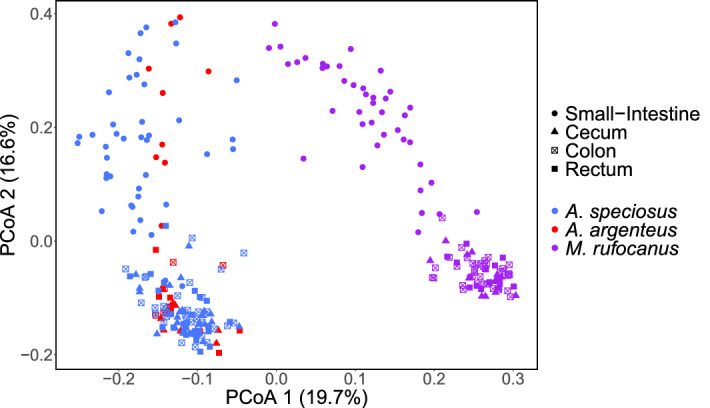
PCoA plot according to unweighted UniFrac in which all samples are plotted. Color indicates host species and shape indicates gut region. The percentages in parenthesis are the proportion of variation explained by the PCoA axis.

**Figure 3:**
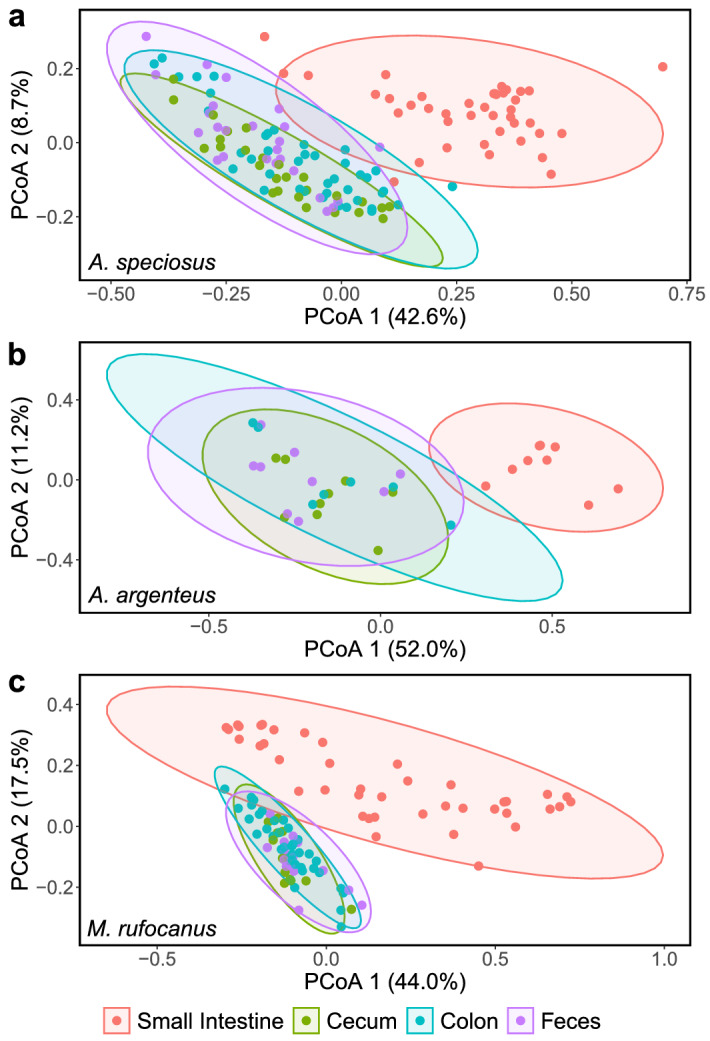
PCoA plots showing among gut region variation of the gut microbiome within (**a**) *A. speciosus*, (**b**) *A. argenteus*, and (**c**) *M. rufocanus* based on weighted UniFrac. Ellipses indicate 95% confidence interval and the percentages in parenthesis are the proportion of variation explained by the PCoA axis.

To further explore changes in the gut bacterial community structure along the GIT, we utilized pairwise PERMANOVAs to determine if each gut region harbored a unique bacterial community. Consistent with our hypothesis, we found that gut region had a highly significant effect when the small intestine was compared to the cecum, colon, or rectum in all three host species regardless of beta diversity metric (PERMANOVA: all *p* < 0.01; Supplementary Tables S15–S17). Furthermore, the effect size was much larger than it was for pairwise comparisons among the three regions of the lower GIT as we expected (Supplementary Tables S15–S17). Indeed, gut region was not always distinguishable among the cecum, colon, and rectum. Specifically, in pairwise comparisons among the three gut regions in *A. speciosus*, gut region had a significant effect based on Bray–Curtis and weighted UniFrac (PERMANOVA: all *p* < 0*.*05; Supplementary Table S15), but not Jaccard or unweighted UniFrac (PERMANOVA: all p > 0.05; Supplementary Table S15). No significant effect was found when the same regions were compared in *A. argenteus* (PERMANOVA: all *p* > 0.05; Supplementary Table S16) while in *M. rufocanus*, gut region significantly impacted beta diversity when the colon and cecum (PERMANOVA: R^2^ = 0.04, F = 2.26, *p* = 0.041) as well as the colon and rectum (PERMANOVA: R^2^ = 0.04, F = 2.35, *p* = 0.41) were compared based on weighted UniFrac alone (Supplementary Table S17). Our PCoA plots showed similar results as samples from the small intestine clustered separate from the others and a large degree of overlap occurred in the clustering of the cecum, colon, and rectum, but not entirely (Figs. 2, 3, Supplementary Figs. S3, S4).

In all three host species, field site had a larger effect than gut region based on R^2^ values for pairwise comparisons among the three regions of the lower GIT (Supplementary Tables S15–S17). For example, when comparing the gut microbiota of the colon and cecum in *A. speciosus* based on weighted UniFrac dissimilarity, the effect of site (PERMANOVA: R^2^ = 0.083, F = 2.217, *p* = 0.013; Supplementary Table S15) was more than twice as large as gut region (PERMANOVA: R^2^ = 0.031, F = 2.449, *p* = 0.04; Supplementary Table S15). When comparing the small intestine to the cecum, colon, or rectum, the effect of gut region was much larger than field site for both *A. speciosus* and *A. argenteus* regardless of dissimilarity metric (Supplementary Tables S15 and S16). However, in *M. rufocanus*, site had a slightly larger effect than gut region for Jaccard (e.g. PERMANOVA of small intestine—cecum; gut region, R^2^ = 0.067, F = 4.09, *p* = 0.001; site, R^2^ = 0.108, F = 2.206, *p* = 0.001; Supplementary Table S17) and Bray–Curtis dissimilarities, but the opposite was true for unweighted UniFrac while site had no effect according to weighted UniFrac distance (e.g. PERMANOVA of small intestine—cecum: gut region, R^2^ = 0.062, F = 1.381, *p* = 0.177; Supplementary Table S17). Age and sex widely effected the gut microbiota beta diversity in both *A. speciosus* and *M. rufocanus*, especially when comparing the three regions of the lower GIT, but no effect was found for *A. argenteus* (Supplementary Tables S15, S17).

### Among host species beta diversity

**Figure 4:**
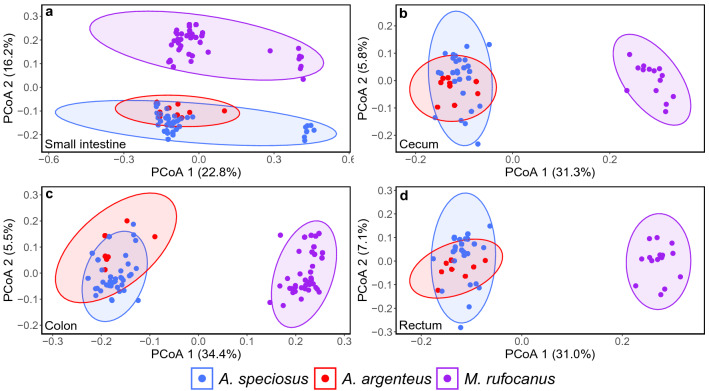
PCoA plots showing among species variation of the gut microbiome within (**a**) the small intestine, (**b**) the cecum, (**c**) the colon, and (**d**) the rectum based on unweighted UniFrac. Ellipses indicate 95% confidence interval and the percentages in parenthesis are the proportion of variation explained by the PCoA axis.

Host species had a significant effect on beta diversity for the small intestine, cecum, colon, and rectum for Jaccard and Bray–Curtis as well as unweighted and weighted UniFrac distances when all three species were included (PERMANOVA: all *p* < 0.05; Supplementary Table S18). Field site also had a significant impact on all gut regions according to Jaccard, Bray–Curtis and unweighted UniFrac (PERMANOVA: all *p* < 0.05; Supplementary Table S18) although the effect size was several times smaller than host species. Sex and age had no effect in any gut region (PERMANOVA: all *p* > 0.05; Supplementary Table S18). The PCoA plots confirmed these findings as there was clustering according to host species within each gut region (Fig. 2, 4, Supplementary Figs. S3, S5). For the small intestine, however, there was a large overlap for weighted UniFrac as well as a small sub-clustering for both *A. speciosus* and *M. rufocanus* that could not be explained by site, age, or sex (Fig. 4).

To test if there were larger differences in the bacterial community structure between *M. rufocanus* and either *Apodemus* spp. than between *A. speciosus* or *A. argenteus*, between species pairwise analyses were utilized (Supplementary Tables S19–S21). We found that host species had a significant effect when the small intestine, cecum, colon, and rectum were compared between *M. rufocanus* and both species of *Apodemus* (PERMANOVA: all *p* < 0.05; Supplementary Tables S19, S21). Furthermore, the effect size was larger when comparing the lower GIT such as the cecum between *A. speciosus* and *M. rufocanus* (PERMANOVA: weighted UniFrac, R^2^ = 0.23, F = 11.786, *p* = 0.001; Supplementary Table S19) than when comparing the small intestine (PERMANOVA: weighted UniFrac, R^2^ = 0.174, F = 17.242, *p* = 0.001; Supplementary Table S19). When compared between *A. speciosus* and *A. argenteus*, host species was significant for Jaccard, Bray–Curtis, and unweighted UniFrac for all gut regions (all *p* < 0.01), but only the cecum based on weighted UniFrac (PERMANOVA: R^2^ = 0.096, F = 3.77, *p* = 0.003; Supplementary Table S20). Importantly, the effect size was smaller than it was when comparing either *Apodemus* species to *M. rufocanus* (Supplementary Tables S19–S21). Field site was also significant for most pairwise comparisons (Supplementary Tables S19–S21) though notably the R^2^ value was smaller than it was for host species in comparisons between *M. rufocanus* and either species of *Apodemus* (Supplementary Tables S19,S21). The opposite was true when comparing *A. speciosus* and *A. argenteus* as site had a slightly larger R^2^ value than host species (Supplementary Table S20). Sex and age were rarely significant in any of the pairwise analyses (Supplementary Tables S19–S21).

### Within host species/among gut region microbiota taxonomic composition

By comparing relative abundances of bacterial genera along the GIT in each host species when all GIT regions were included using LEfSe analysis, we found *Ruminococcaceae* NK4A21 group had significantly higher relative abundance in the rectum of *A. speciosus* and *Treponema* 2 was higher in the cecum of *M. rufocanus* (Supplementary Tables S22, S24). Four genera in the small intestine, 13 in the cecum, five in the colon, and 17 in the rectum of *A. argenteus* were found to exhibit significantly higher abundance, suggesting highly differential microbiome communities along the length of the GIT (Supplementary Tables S22, S24). It must be noted that the relatively larger number of significant differences in microbial abundances in *A. argenteus* may be a type 1 error due to the small sample size (nine individuals).

**Figure 5:**
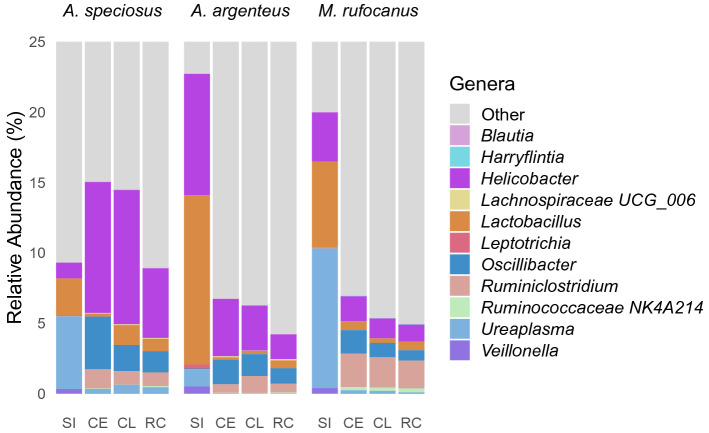
Relative abundances of bacterial genera exhibiting either a similar trend along the GIT of all three rodent species or a species-specific trend. *SI* small intestine, *CE* cecum, *CL* colon, and *RC* rectum.

To develop a clearer picture regarding differential relative abundance of the various bacterial genera within the four gut regions, pairwise LEfSe analysis was conducted (Supplementary Tables S23, S25–S30). We were specifically interested if relative abundances in the small intestine were distinctly different than the cecum, colon, or rectum, as well as if there was a high degree of similarity throughout the lower GIT. We found a large number of bacterial genera with significantly different relative abundances between the small intestine and the lower GIT in all three host species with more genera exhibiting higher relative abundance in the lower GIT as compared to the small intestine than vice versa (Supplementary Table S23). Some bacterial genera such as *Lactobacillus* and *Veillonella* were found to have higher relative abundance within the small intestine in all three host species regardless of which region of the lower GIT it was compared to (Fig. 5, Supplementary Tables S25, S27, S29). Others such as *Leptotrichia* had higher relative abundance within the small intestine as compared to the cecum, colon, or rectum in both *Apodemus *spp. but not in *M. rufocanus* (Fig. 5, Supplementary Tables S25, S27, S29). Many exhibited a host species-specific trend such as the higher relative abundance of *Helicobacter* in the small intestine of *M. rufocanus* as compared to the lower GIT (Fig. 5, Supplementary Table S29). Similarly, some genera were found to have higher relative abundances throughout the lower GIT as compared to the small intestine in all three host species such as *Oscillibacter* and *Ruminiclostridium* (Fig. 5, Supplementary Tables S25, S27, S29). However, most bacterial genera either exhibited higher relative abundance throughout the lower GIT (compared to the small intestine) in a single host species such as *Harryflintia* in *M. rufocanus* or no clear trend was found (Fig. 5, Supplementary Tables S25, S27, S29).

Relatively few microbial genera exhibited differential relative abundance when comparing the cecum, colon, and rectum, agreeing with our hypothesis (Supplementary Tables S23, S26, S28, S30). Disagreeing with our predictions, in both *A. speciosus* and *M. rufocanus* relative abundances were found to be more similar between the rectum and cecum than the rectum and colon where 7 and 12 (*A. speciosus* and M. *rufocanus* respectively) microbial genera were found to have significantly higher relative abundance in the rectum as compared to the colon (Supplementary Tables S23, S26, S30). Notably, there was little consistency in terms of which bacterial genera were found to exhibit higher relative abundance in each of the lower gut regions (Supplementary Tables S26, S28, S30). However, higher relative abundance of *Oscillibacter* was found in the cecum of both *A. speciosus*, and *M. rufocanus* as compared to the colon and rectum (Supplementary Tables S26, S30), but not in *A. argenteus* (Supplementary Table S28). Furthermore, *Ruminococcus* 1 and *Pygmaiobacter* in the rectum of *M. rufocanus* (Supplementary Table S30), *Ruminococcaceae* NK4A214 in the rectum of *A. speciosus* (Fig. 5, Supplementary Table S26), and *Rodentibacter* in the rectum of *A. argenteus* (Supplementary Table S28) were found to have higher relative abundance as compared to the colon or cecum.

When exploring the effect of host species on the relative abundance of bacterial genera within each gut region using LEfSe analysis with all three host species included (i.e. non-pairwise), we found a large number of genera exhibiting host species-specific higher relative abundance (Supplementary Tables S31, S33). To determine if the relative abundance of bacterial taxa were more similar between *A. speciosus* and *A. argenteus* than between *M. rufocanus* and either species of *Apodemus*, a pairwise LEfSe analysis between each host species for each gut region was utilized (Supplementary Tables S32, S34–S37). The largest number of variations in relative abundances were between *M. rufocanus* and both species of *Apodemus* as predicted (Supplementary Tables S32, S34–S37). Far fewer genera were found to have higher relative abundance in either *A. speciosus* or *A. argenteus* when compared to each other (Supplementary Tables S32, S34–S37). Some bacterial genera showed a similar trend when comparing *M. rufocanus* to *A. speciosus* or *A. argenteus*. For example, there was higher relative abundance of *Ruminococcaceae* NK4A214 throughout the lower GIT of *M. rufocanus* (Fig. 5, Supplementary Tables S35–S37) as well as *Ureaplasma* within the small intestine when compared to either *A. speciosus* or *A. argenteus* (Supplementary Table S34). Similarly, *Lachnospiraceae* UCG_006 was found to have higher relative abundance throughout the lower GIT in both *A. speciosus* and *A. argenteus* as compared to *M. rufocanus* (Fig. 5, Supplementary Tables S35–S37).

## Discussion

Our understanding of the role that host species and gut region plays in shaping the microbial community structure along the GIT within wild populations of animals is limited. In the present study we compared the gut microbiome among four gut regions in three species of sympatric rodents. We investigated whether the microbial community structure was more similar among the cecum, colon, and rectum than between the lower GIT and small intestine. We also tested if larger differences could be found between *M. rufocanus* and *A. speciosus* or *A. argenteus* than between either species of *Apodemus*.

### Gut microbial community structure varies along the GIT

We found a similar trend in gut microbiome diversity along the digestive tract in our three host species as alpha diversity was lowest in the small intestine, but nearly identical in the cecum, colon, and rectum (Fig. 1, Supplementary Fig. S2, Supplementary Tables S4–S7). Our PCoA plots demonstrated a similar pattern with a high degree of overlap in community structure within the lower GIT but was distinct within the small intestine (Fig. 2, 3, Supplementary Figs. S3, S4). This is common among hind gut fermenting animals such as many rodents^10,14,15^ and reptiles^12,23^. In mammals, the transit time of gut content through the small intestine is 10 times faster than through the cecum or colon^24^. This has led some to postulate that only those bacterial species that are able to adhere to the mucosal wall can become established while the rest pass into the lower GIT^13^, thereby limiting the number of bacterial species residing in the region permanently as well as their abundances. Furthermore, the host immune system has a higher level of activity within the small intestine as compared to the cecum or colon through the secretion of antimicrobial peptides by Paneth cells in the epithelial wall^7^. This not only helps defend against pathogens, but also shapes the microbial community by restricting which bacterial species can successfully colonize the mucosa, many of which are beneficial for the host. For example, in all three species we found significantly higher abundance of the presumably probiotic genus *Lactobacillus* within the small intestine (Fig. 5, Supplementary Tables S25, S27, S29), not an uncommon finding^14,15^.

Our pairwise PERMANOVA analyses suggests some variability in the gut microbial community structure along the lower GIT of both *A. speciosus* and *M. rufocanus* although the effect of site was larger than gut region (Supplementary Tables S15–S17). Geographic distance between locales can impact the gut microbial community structure of mammals due to dispersal limitation of bacteria and could partially explain our findings^8^. On the other hand, because the distance between sites in our study is relatively small, a more likely explanation is the consumption of different food items which has a profound impact on the microbial composition of the GIT, especially the lower GIT^11,13,25^. Site-specific dietary differences could result from variation in plant and insect communities (food items) or altered competition for resources due to a difference in rodent community structure (Supplementary Table S1). There is overlap in the physiology and functionality of the different regions of the lower GIT, thereby requiring a similar gut microbiota^13,26^ that is likely to respond in a similar fashion to dietary changes.

While no differences in relative abundance were found for the majority of microbial genera among the different gut regions of the lower GIT based on pairwise LEfSe analysis, there were several exceptions such as *Ruminococcous* 1 in the rectum of *M. rufocanus* or *Ruminococcaceae* NK4A214 in the rectum of *A. speciosus* (Fig. 5, Supplementary Tables S26, S28, S30). This partially supports the results of PERMANOVA (Supplementary Tables S15–S17) suggesting that differences in community structure are driven by a few highly abundant taxa within each gut region. While the high degree of similarity in abundances between the cecum and colon is of no surprise due to their overlapping physiological functionality, they are not the same. For example, the cecum’s main role is fermentation and energy absorption while reabsorption of water occurs in the colon before defecation causing the physical environments to differ^13,26^ possibly explaining the differential abundances observed. Alternatively, micro-geographic differences in the microbiome can occur within the same gut region, thereby reducing the accuracy of our characterization and falsely discovering differences as we did not sample the entirety of each region^27^. But our sampling technique which took gut content from a large portion of each gut region, as opposed to a biopsy, as well as our large sample size should control for this. Therefore, we believe that when only interested in presence or absence of bacteria within the lower GIT, sampling from the cecum, colon, or rectum is appropriate. Furthermore, because the rectum samples were fecal matter collected before defecation, thereby controlling for environmental bacterial contaminants, feces may be an appropriate tool for non-invasive sampling when studying the microbial communities of the lower GIT in these host species. However, caution should be taken when bacterial abundances are of concern or when interested in the microbial community of the small intestine.

### Host species distinguishability in the gut microbiome

Not only was the gut microbiome distinct among the more distantly related vole and the two field mice in all four gut regions, there were also differences between *A. speciosus* and *A. argenteus*. Importantly, we found that host species explained more of the variation than field site, similar to what was reported by Knowles et al.^28^. While some of the variation is potentially attributable to host species-specific immunity^29^ or mucus characteristics^13,30^, dietary preferences are likely one of the main factors especially within the lower GIT. The cecum, and to a lesser extent the colon, is important for the breakdown of plant polysaccharides in hind gut fermenting animals, especially herbivorous species such as *M. rufocanus*^31^. We found that alpha diversity of the cecum, colon, and rectum was highest within *M. rufocanus* (Fig. 1, Supplementary Fig. S2, Supplementary Tables S8–11) as is typically seen in herbivores as compared to either omnivores or carnivores^32^. Furthermore, many of the relatively more abundant genera in the lower gut such as *Ruminococcaceae* NK4A214 and *Ruminiclostridium* in *M. rufocanus*, *Lachnospiraceae* UCG_006 from both *A. speciosus* and *A. argenteus*, *Streptococcus* in *A. speciosus*, and *Prevotellaceae* UCG_003 in *A. argenteus* are known fermenters of various food materials^33–36^ (Supplementary Fig. 5, Supplementary Tables S35–S37).

The gut microbiota was less distinguishable between *A. speciosus* and *A. argenteus* during pairwise analysis where site had a larger effect. These species share a large degree of overlap in dietary preferences^22^, thereby bringing about more similarity in their gut microbial community structure as compared to *M. rufocanus*^11^. While the portion of their diet that is not shared (e.g. *A. speciosus*’ preference for walnuts) may account for the observed differences, another explanation could be exposure to different environmental bacteria in their micro-habitat specific environments as *A. argenteus* is more arboreal^19^. Alternatively, gut bacteria are known to affect the metabolic rate of the host and provide blood metabolites the host is unable to synthesize itself^37^. Because *A. argenteus* has double the basal metabolic rate of *A. speciosus*^38^, it may require the presence of bacterial species that help meet the higher energy requirements. It must be noted that the small sample size of *A. argenteus* might be causing a false identification of a differential microbial community structure between *Apodemus* spp. that a larger and more even sample size would not.

Interestingly, there was no difference in Faith’s PD within the small intestine in any of the three species (Fig. 1, Supplementary Table S9), nor in the number of ASVs between *A. speciosus* and *A. argenteus* (Supplementary Fig. S2, Supplementary Table S11). There was also a high degree of overlap in the microbial community structure based on the weighted UniFrac PCoA plots (Supplementary Figs. S3, S5). This suggests more among host species similarity in the gut microbiota within the small intestine than the lower GIT. Environmental factors such as low pH and fast transit times may induce similar evolutionary pressures on the bacteria within the small intestine of hind gut fermenters such as those in this study^13,24^ thereby providing an initial across host species filter before host-bacteria co-evolution can occur with those bacteria that remain. If the microbial taxonomic groups that can survive in such an environment are limited, then the relative phylogenic relatedness of the resident bacterial species may be similar within each host species explaining the lack of significant results for Faith’s PD as well as the overlap in the weighted UniFrac PCoA plots. However, if this trend was driven entirely by species composition, we would expect to see similar results for the unweighted UniFrac PCoA plot. These results were also largely unsupported by the results of our PERMANOVAs.

The small intestine is less important for digestion than the lower GIT within hindgut fermenters^10,13^ so diet may play a diminished role in shaping the gut microbiota within it. While the mammalian immune system is partially conserved among species it is not identical^39,40^. Because immunological activity is highest within the small intestine and helps shape the gut microbiota^7^, species-specific differences such as the production of differential anti-microbial peptides or expression of various isotypes of Immunoglobulin A may explain the observed differences^26,40^. Further research must be conducted as few studies characterizing the impact of the host immune system on the gut microbiota in species other than humans and lab mice have been conducted^39,40^.

We found multiple genera to have significantly higher relative abundance within the small intestine of each host species as compared to the others. In particular, we found that the probiotic genus *Lactobacillus* had the highest relative abundance in *A. argenteus* and least abundance in *A. speciosus.* However, this was likely caused by a single individual of *A. argenteus* within our small sample size with 75% of sequences identified as *Lactobacillus* while only 4% on average were in the remaining eight individuals. Furthermore, while the majority of *Lactobacillus* sequences were of an unidentified species, we also found host species-specific bacterial species such as *L. gasseri* in 26% of *A. speciosus* and *L. rodentium* in 72% of *M. rufocanus*. Perhaps this is due to host selection as these sympatric species of rodent would likely have been exposed to many of the same bacteria due to an overlap in ecological traits such as dietary niche.

## Conclusion

By sampling the gut microbiome in multiple locations across the GIT, we were able to show that the microbial community within the small intestine is distinct from the lower GIT in three sympatric species of wild rodents, similar to previous studies^10,14,15^. Within the lower GIT, there was no difference in alpha diversity among the cecum, colon, nor rectum, and rarely did gut region affect beta diversity except when both phylogenetic relatedness and abundance were considered (i.e. weighted UniFrac analysis). Furthermore, few genera were found to differ in relative abundance along the lower GIT as opposed to the large number of more abundant genera found when comparing the upper and lower GIT. Therefore, feces may be appropriate for studying the gut microbiota of the lower GIT if caution is taken regarding the relative abundances of bacterial genera but should not be utilized for investigating the small intestine. We did not test for phylosymbiosis directly, but we found that each species harbored a unique microbiome within each gut region, especially when comparing the distantly related *M. rufocanus* to both *A. speciosus* and *A. argenteus*. The main driver for these differences is likely due to dietary niche as many of the genera that differed in relative abundance are known to aid in digestion of plant polysaccharides within the lower GIT. In this study, the small intestine microbiome may be more similar among species than the lower GIT based on weighted UniFrac PCoA and Faith’s PD, perhaps suggesting similar evolutionarily pressures acting on bacteria across host species. However, this was not confirmed by PERMANOVA. Future research identifying specific ecological (e.g. dietary preference), physiological (e.g. gut transit times), or immunological traits that may explain both similarities and differences in the gut microbiome profiles of wild sympatric species must be conducted.

## Methods

### Host species and field sampling

Field sampling was conducted in October 2019 at four field sites (Shirakkeyama, Chitoseyama, Harushinai, and Mukoyama) within the Kamikawa Chubu National Forest in central Hokkaido, Japan (Supplementary Fig. S1, Supplementary Table S1) using Sherman traps baited with oatmeal. Traps were set 10 m apart in a 4 × 10 grid pattern with two trap grids at each site except for Shirakkeyama which contained three. Furthermore, due to constraints of the terrain at Mukoyama, the trap grids utilized were 2 × 20. Traps were checked within one hour after sunrise for two or three consecutive days at each site. Any trap containing an animal was replaced with a fresh trap and all alive individuals were transported to the department of parasitology at Asahikawa Medical University, Asahikawa, Japan for processing.

### Gut content sampling

After euthanization by cervical dislocation, body weight (g) and sex were recorded. Body weight was used to classify each individual as adult or sub-adult based on average body weight at maturation of each species^41,42^. The entire digestive tract was removed and segmented into three parts corresponding to the small intestine, cecum, and large intestine. Using a small steel spatula (2 mm width), feces was collected directly from the rectum to avoid environmental contamination, and gut content was collected from the ileum within the small intestine, the central part of the cecum, and the ascending colon. All samples were collected in sterile 2 ml vials and placed in a – 80 °C freezer within one hour after collection. All tools were flame sterilized and all surfaces sterilized with 10% bleach followed by 70% ethanol before each use.

### DNA extraction, PCR amplification, and amplicon sequencing

All gut content and fecal samples were placed on dry ice and transported to the Laboratory of Parasitology in the Faculty of Veterinary Medicine at Hokkaido University, Sapporo, Japan (two-and-a-half-hour journey), and immediately placed into a – 80 °C freezer upon arrival. DNA extraction was performed using the QIAamp fast DNA Stool Mini Kit (Qiagen) after bead beating with four 3 mm beads and 1 mg of 0.1 mm beads per sample following Hayakawa et al.^43^. A negative control was included with each batch of 24 samples to determine potential contaminants introduced during processing. The V3–V4 region of the 16S rRNA gene was amplified by PCR using universal primers 341F-805R^44^ in a solution recommend by Illumina with a slight modification and consisting of 0.5 µl of each primer at a concentration of 10 µM, 12.5 µl of 2 × Kapa HiFi HotStart Ready Mix (KAPA Biosystems), 9 µl of molecular water, and 2.5 µl of extracted DNA from gut content and fecal samples. The following thermocycler conditions were used: initial denaturation at 95 °C for 3 min, then 25 cycles of denaturation at 95 °C, annealing at 55 °C, and extension at 72 °C for 30 s each, followed by a final extension at 72 °C for 5 min. An additional negative control was included in each PCR. Both DNA extraction and PCR were performed under sterile conditions within a biosafety cabinet in which all equipment and surfaces were cleaned with 70% ethanol and sterilized for 20 min by UV light before use. Following the manufactures instructions, library preparation was performed using Nextera XT DNA Index Kit v2 set A, B, C, or D, and sequenced on an Illumina MiSeq 300 bp paired-end platform using a v3 Reagent Kit. Raw sequence reads were submitted to the DNA database of Japan (DDBJ) with the accession number DRA011343.

### Analyses

The standard DADA2 denoising pipeline^45^ in Qiime2 version 2020.2^46^, was utilized for demultiplexing, merging forward and reverse paired-end reads, quality filtering, and removal of chimeric sequences to produce a feature table of Amplicon Sequence Variants (ASVs). Because the number of sequence reads within the majority of negative control samples were exceedingly low (average = 131 ± 171 SD), most could not be used as a reference for decontaminating our samples by the prevalence method within the decontam package^47^ in R version 4.0.2^48^ and was therefore unreliable. Instead, we used the frequency method with a threshold of 0.1 and the frequency distribution plots of all 147 potential contaminant sequences were checked for confirmation. Furthermore, the negative controls were manually checked for the potential contaminant sequences and two (uncultured Rickettsiales and Clostridiales vadinBB60 group) were found to be highly prevalent with high abundance in our samples but non-existent within our negative controls. In total 145 contaminant sequences were removed from the ASV table. Taxonomic classification^49^ of the decontaminated sequences was assigned using SILVA classifier (release 132). ASVs identified as Archaea, Eukaryota, mitochondria, and Chloroplastida were removed as well as bacterial sequences not assigned to phylum level. A rooted phylogenetic tree was then generated using the FastTree method in Qiime2^50^.

All samples were rarefied to a sampling depth of 10,000 reads for diversity analysis to cover most of the bacterial community within all gut regions according to alpha rarefaction analysis, leading to the exclusion of three samples (two small intestine and one rectum from *M. rufocanus*) due to low sequence read counts. Microbial diversity was quantified using four α-diversity measurements (Shannon diversity, Faith’s phylogenetic diversity (PD), Pielou’s evenness, and number of ASVs) and four β-diversity metrics (Jaccard dissimilarity, Bray–Curtis dissimilarity, unweighted UniFrac, and weighted UniFrac) in Qiime2. To determine significant differences in α-diversity among the different gut regions within each species as well as the same gut region among species, a generalized linear mixed effects model (GLME) with gaussian distribution was utilized. The response variable was log transformed alpha diversity with host species (or gut region), sex, and age (i.e. adult/sub-adult) as fixed effects, and field site as the random effect as performed in R using the nlme package version 3.1–150^51^. To determine the effect of host species or gut region on community structure of the gut microbiome, we first visualized the data using principle coordinate analysis (PCoA) plots based on the four beta diversity metrics using the R package phyloseq^52^. We then analyzed the effect of field site, sex, and age (adult/sub-adult) on β-diversity, by using permutational multivariate analysis of variance (PERMANOVA) with 999 permutations and the by = “margin” option to account for all variables using the adonis2 function in the vegan package in R^53^. We first included all gut regions for each species or all three species for among species comparison of each gut region before running a series of pairwise comparisons. Because all nine *A. argenteus* were classified as adults, age was not included as a variable for within species (i.e. among gut region) GLME or PERMANOVA.

To determine differences in relative abundances of microbial genera, we ran linear discriminant analysis effect size (LEfSe) using the Huttenhower lab Galaxy pipeline^54^. We first ran the analysis while including all GIT regions for each species in which gut region (i.e. small intestine, cecum, colon, and rectum) was the class and host ID the subject to analyze within species variation. We then analyzed among species variation of each gut region including all three host species in which the class was host species. Pairwise analysis was then conducted between each gut region within each host species (e.g. small intestine vs. colon in *A. speciosus*), as well as each gut region between each species (e.g. cecum in *A. speciosus* vs. *M. rufocanus*).

### Ethical statement

Permission for the collection of animals was given by the Hokkaido prefectural government (permit numbers 271 and 272), Kamikawa Chubu national forest Asahikawa office (permit number 422), the city of Asahikawa municipal office (permit number 275), and the city of Biei municipal office (permit number 26). Experimental design and handling of animals was approved and carried out in accordance with the guidelines established by the Institutional Animal Care and Use Committee of the National University Corporation Hokkaido University (reference number 15-0121). This study was carried out in compliance with the ARRIVE guidelines.

## Supplementary Information

Supplementary Information.
