# Supplementary material for: Comparing the gut microbiome along the gastrointestinal tract of three sympatric species of wild rodents
Source: Sci Rep. 2021 Oct 7;11:19929. doi: 10.1038/s41598-021-99379-6 (PMC8497572; doi:10.1038/s41598-021-99379-6)
Supplement: Supplementary file 1 — Supplementary Information. [file 41598_2021_99379_MOESM1_ESM.pdf]

TITLE: Comparing the gut microbiome along the gastrointestinal tract of three sympatric species of wild rodents

AUTHORS: Jason L. Anders, Mohamed Abdallah Mohamed Moustafa, Wessam Mohamed Ahmed Mohamed, Takashi Hayakawa, Ryo Nakao, Itsuro Koizumi

Supplemental Results

Most abundant phyla within each gut regions and host species

Firmicutes, Bacteroidetes, and Epsilonbacteraeota were the most abundant phyla in the cecum, colon, and rectum, of *A. speciosus* and *A. argenteus* accounting for 85 to 95 percent of all microbes, while Firmicutes, Bacteroides, and Spirochaetes were dominant in *M. rufocanus,* although relative abundances of each varied depending on host species and gut region. In the small intestine of *M. rufocanus* the four most abundant phyla were Bacteroides (40.8%), Proteobacteria (20.1%), Firmicutes (18.5%), and Tenericutes (14.8%; Fig. 4). Firmicutes (55.7%), Tenericutes (15.5%), Bacteroides (13.4%), and Proteobacteria (12.4%) made up the largest portion of the microbiome in the small intestine of *A. speciosus* and the most dominant in *A. argenteus* were Tenericutes (26.4%), Proteobacteria (24.2%), Firmicutes (19.9%), and Spirochaetes (15.4%). Notably, Tenericutes and Proteobacteria had higher relative abundance in the small intestine in all three host species as compared to the cecum, colon, or rectum.

SUPPLEMENTARY FIGURES

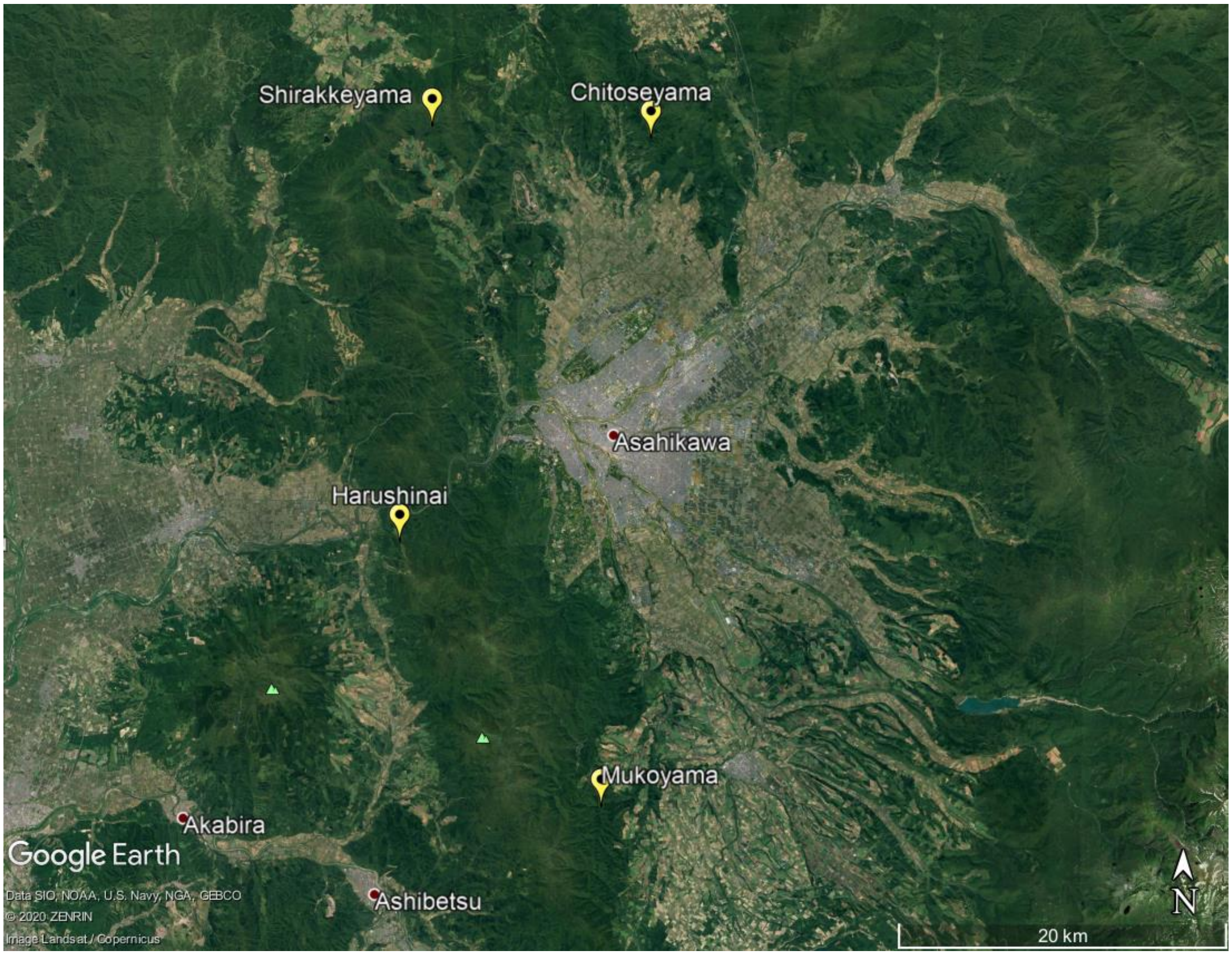

Fig. S1: Satellite image of field sites within Kamikawa Chubu National Forest in central Hokkaido. The map was created using Google Earth Pro (version 7.3.3, https://www.google.com/earth/versions/). Map data: Google, SIO, NOAA, U.S. Navy, NGA, GEBCO, © 2020 ZENRIN Image Landsat/ Copernicus

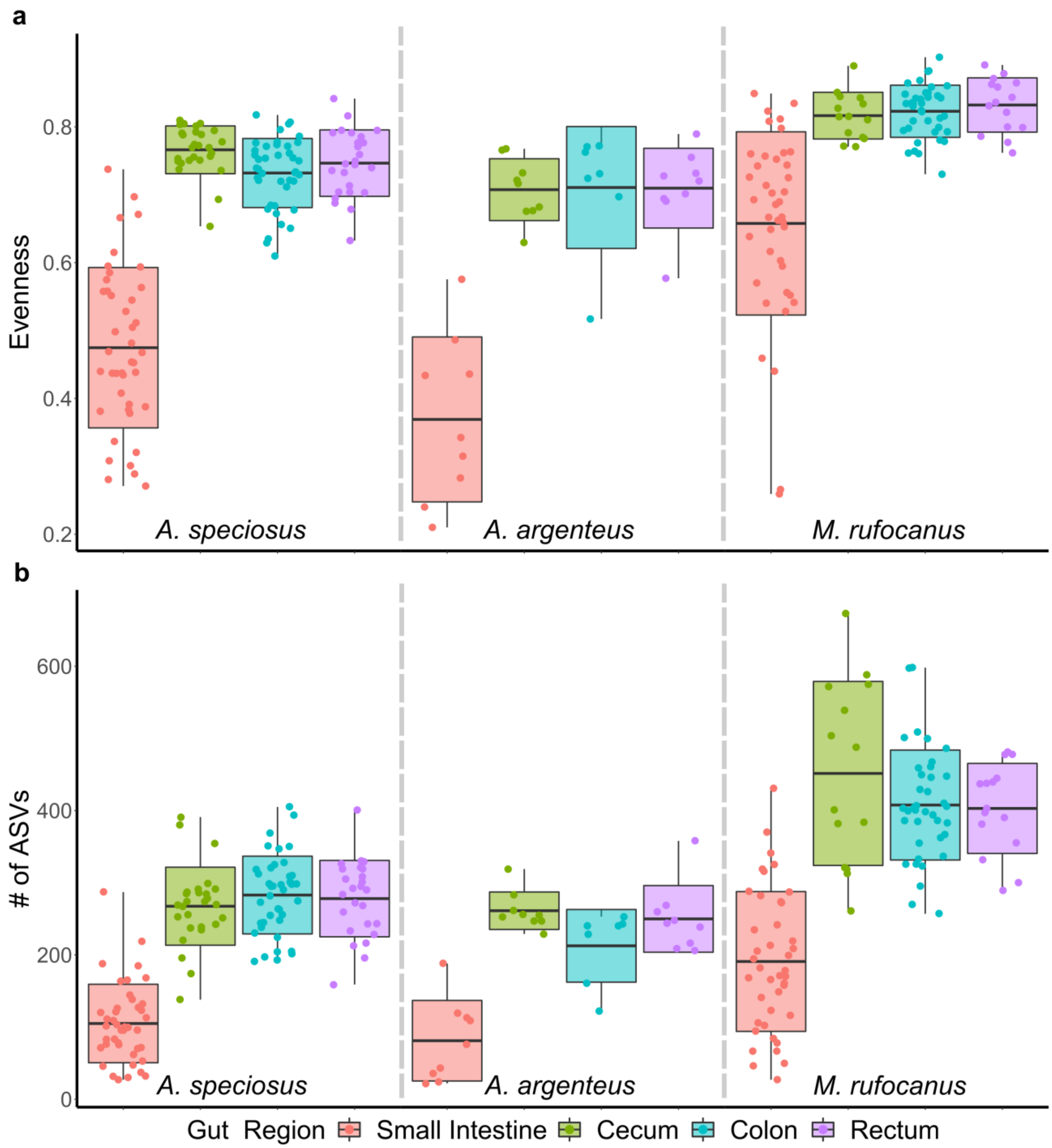

Fig. S2: Alpha diversity within each gut region of each species based on a) Pielou's evenness and b) number of ASVs. Dashed lines separate host species.

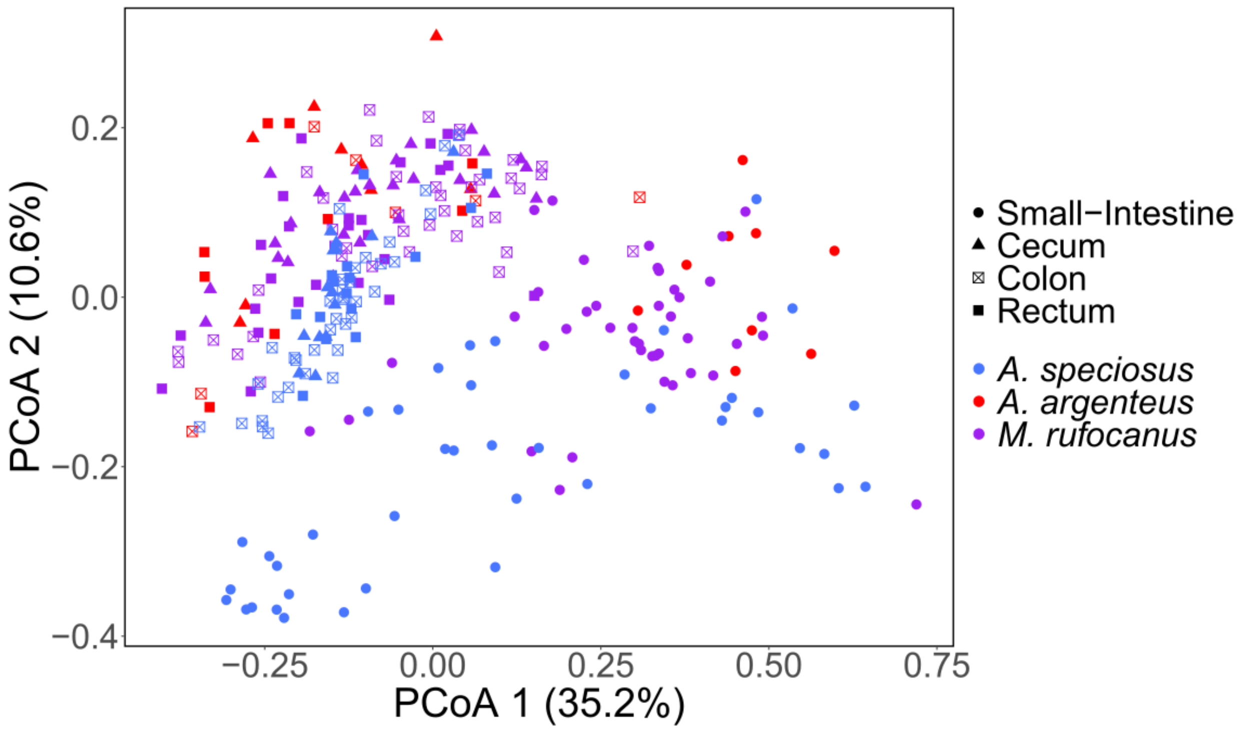

Fig. S3: PCoA plot according to weighted UniFrac in which all samples are plotted. Color indicates host species and shape indicates gut region. The percentages in parenthesis is the proportion of variation explained by the PCoA axis.

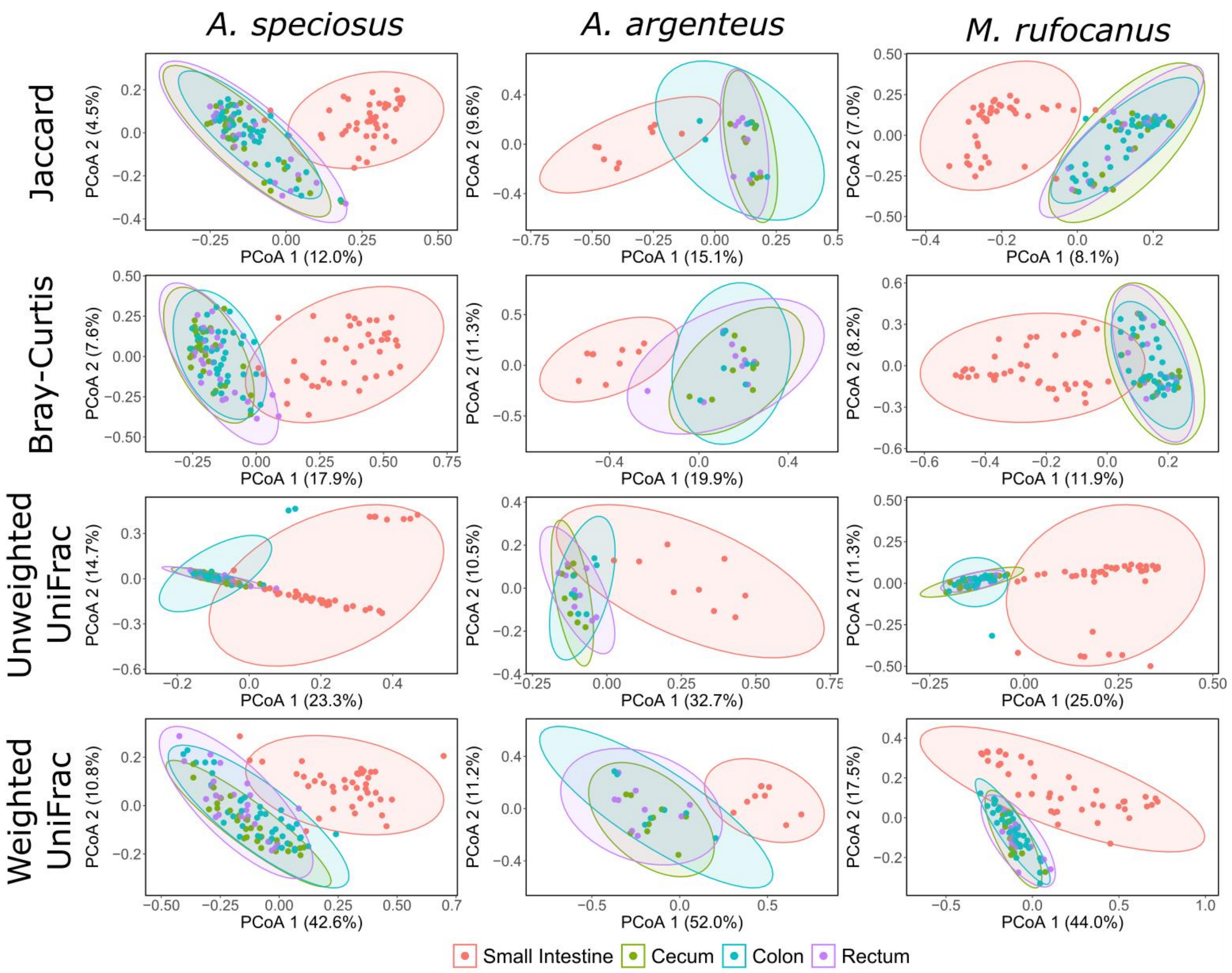

Fig. S4: Variation in the gut microbiome among gut regions within each species where column is host species and row is distance metric. Orange is the small intestine, green is cecum, light turquoise is colon, and purple is rectum. Ellipses indicate 95% confidence interval and the percentages in parenthesis is the proportion of variation explained by the PCoA axis.

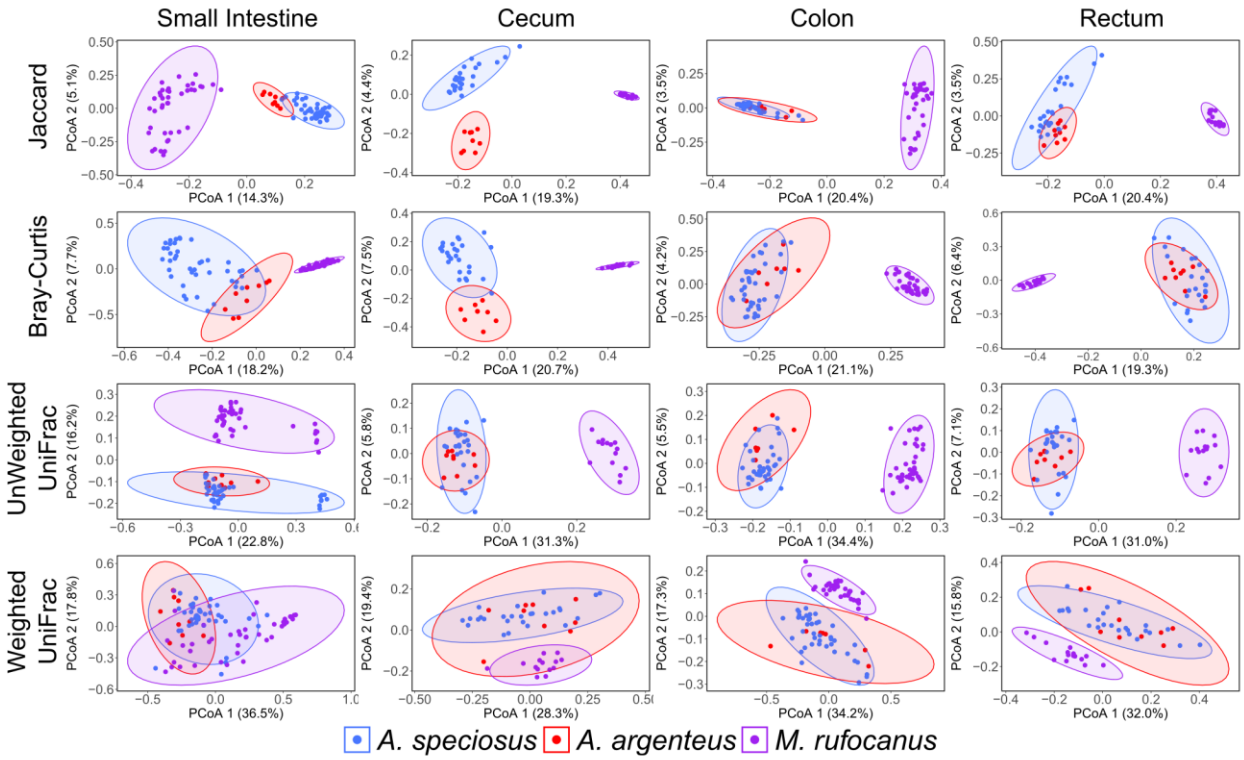

Fig. S5: Variation in the gut microbiome among species within each gut region where column is gut region and row is distance metric. Blue is *A. speciosus*, red is *A. argenteus*, and purple is *M. rufocanus*. Ellipses indicate 95% confidence interval, and the percentages in parenthesis is the proportion of variation explained by the PCoA axis.

SUPPLEMENTARY TABLES

Field site information

Site	Latitude	Longitude	*A. speciosus*	*A. argenteus*	*M. rufocanus*
Chitoseyama	N: 43° 56' 30.64"	E: 142° 24' 10.09"	6	0	16
Harushinai	N: 43° 42' 19.82"	E: 142° 12' 08.28"	5	0	20
Mukoyama	N: 43° 34' 07.41"	E: 142° 21' 34.57"	11	5	3
Shirakkeyama	N: 43° 56' 14.15"	E: 142° 13' 21.56"	20	4	4
**Total**			42	9	43

Table S1: Coordinates of each field site and the number of individuals of each species captured at each.

Number of gut content/fecal matter samples per gut region

Species	Small Intestine	Cecum	Colon	Rectum
A. speciosus	42	27	41	25
A. argenteus	9	9	7	9
M. rufocanus	43	14	38	16
**Total**	94	50	86	50

Table S2: The number of gut content or fecal matter (rectum only) samples per gut region per host species.

Average number of reads per sample

Species	All	Small Intestine	Cecum	Colon	Rectum
A. speciosus	45406 ± 1004	51095 ± 1571	35048 ± 2310	48865 ± 1301	46763 ± 2174
A. argenteus	46650 ± 2600	34063 ± 5044	55879 ± 3700	38635 ± 3980	56242 ± 2960
M. rufocanus	39956 ± 1543	41959 ± 3099	45232 ± 4857	39674 ± 1649	30623 ± 2636

Table S3: Average number of sequences per sample for each gut region in each species plus or minus standard error of the mean.

Shannon diversity among gut region (LME results)

Species	Gut Region	Small Intestine Est.	Small Intestine SE	Small Intestine *p*	Cecum Est.	Cecum SE	Cecum *p*	Colon Est.	Colon SE	Colon *p*	Rectum Est.	Rectum SE	Rectum *p*
A. speciosus	Small intestine	–	–	–	-0.729	0.05	< **.001**	-0.688	0.044	< **.001**	-0.714	0.051	< **.001**
	Cecum	0.729	0.05	< **.001**	–	–	–	0.042	0.05	0.409	0.015	0.056	0.786
	Colon	0.688	0.044	< **.001**	-0.042	0.05	0.409	–	–	–	-0.026	0.051	0.608
	Rectum	0.714	0.051	< **.001**	-0.015	0.056	0.786	0.026	0.051	0.608	–	–	–
	Sex (male)	0.133	0.036	< **.001**	0.133	0.036	< **.001**	0.133	0.036	< **.001**	0.133	0.036	< **.001**
	Age	0.065	0.043	0.136	0.065	0.043	0.136	0.065	0.043	0.136	0.065	0.043	0.136
A. argenteus	Small intestine	–	–	–	-1.006	0.11	< **.001**	-0.956	0.118	< **.001**	-0.998	0.11	< **.001**
	Cecum	1.006	0.11	< **.001**	–	–	–	0.05	0.118	0.675	0.008	0.11	0.941
	Colon	0.956	0.118	< **.001**	-0.05	0.118	0.675	–	–	–	-0.042	0.118	0.726
	Rectum	0.998	0.11	< **.001**	-0.008	0.11	0.941	0.042	0.118	0.726	–	–	–
	Sex (male)	-0.302	0.081	< **.001**	-0.302	0.081	< **.001**	-0.302	0.081	< **.001**	-0.302	0.081	< **.001**
	Age	–	–	–	–	–	–	–	–	–	–	–	–
M. rufocanus	Small intestine	–	–	–	-0.004	0.083	0.963	-0.423	0.05	< **.001**	-0.448	0.068	< **.001**
	Cecum	0.451	0.07	< **.001**	-0.028	0.07	0.69	0.028	0.07	0.69	0.004	0.083	0.963
	Colon	0.423	0.05	< **.001**	–	–	–	–	–	–	-0.024	0.068	0.722
	Rectum	0.448	0.068	< **.001**	-0.451	0.07	< **.001**	0.024	0.068	0.722	–	–	–
	Sex (male)	0.03	0.045	0.508	0.03	0.045	0.508	0.03	0.045	0.508	0.03	0.045	0.508
	Age	-0.044	0.053	0.405	-0.044	0.053	0.405	-0.044	0.053	0.405	-0.044	0.053	0.405

Table S4: Linear mixed effects model results for Shannon diversity comparing gut region within each species, values rounded to the thousandth place. Column is the response variable, rows are explanatory variables. Bold text indicates statistical significance.

Faith’s PD among gut region (LME results)

Species	Gut Region	Small Intestine Est.	Small Intestine SE	Small Intestine *p*	Cecum Est.	Cecum SE	Cecum *p*	Colon Est.	Colon SE	Colon *p*	Feces Est.	Feces SE	Feces *p*
A. speciosus	Small intestine	–	–	–	-0.289	0.052	< **.001**	-0.344	0.046	< **.001**	-0.327	0.054	< **.001**
	Cecum	0.289	0.052	< **.001**	–	–	–	-0.056	0.053	0.294	-0.039	0.058	0.507
	Colon	0.344	0.046	< **.001**	0.056	0.053	0.294	–	–	–	0.017	0.054	0.755
	Rectum	0.327	0.054	< **.001**	0.039	0.058	0.507	-0.017	0.054	0.755	–	–	–
	Sex (male)	0.106	0.038	**0.006**	0.106	0.038	**0.006**	0.106	0.038	**0.006**	0.106	0.038	**0.006**
	Age	0.013	0.047	0.784	0.013	0.047	0.784	0.013	0.047	0.784	0.013	0.047	0.784
A. argenteus	Small intestine	–	–	–	-0.406	0.083	< **.001**	-0.31	0.088	**0.001**	-0.405	0.083	< **.001**
	Cecum	0.406	0.083	< **.001**	–	–	–	0.096	0.088	0.287	0.0004	0.083	0.996
	Colon	0.31	0.088	**0.002**	-0.096	0.088	0.287	–	–	–	-0.096	0.088	0.289
	Rectum	0.405	0.083	< **.001**	-0.0004	0.083	0.996	0.096	0.088	0.289	–	–	–
	Sex (male)	-0.176	0.061	**0.007**	-0.176	0.061	**0.007**	-0.176	0.061	**0.007**	-0.176	0.061	**0.007**
	Age	–	–	–	–	–	–	–	–	–	–	–	–
M. rufocanus	Small intestine	–	–	–	-0.389	0.066	< **.001**	-0.293	0.047	< **.001**	-0.331	0.064	< **.001**
	Cecum	0.389	0.066	< **.001**	–	–	–	0.096	0.067	0.155	0.058	0.078	0.459
	Colon	0.293	0.047	< **.001**	-0.096	0.067	0.155	–	–	–	-0.038	0.065	0.559
	Rectum	0.331	0.064	< **.001**	-0.058	0.078	0.459	0.038	0.065	0.559	–	–	–
	Sex (male)	0.021	0.043	0.628	0.021	0.043	0.628	0.021	0.043	0.628	0.021	0.043	0.628
	Age	-0.07	0.051	0.17	-0.07	0.051	0.17	-0.07	0.051	0.17	-0.07	0.051	0.17

Table S5: Linear mixed effects model results for Faith’s phylogenetic diversity comparing gut region within each species, values rounded to the thousandth place. Column is the response variable, rows are explanatory variables. Bold text indicates statistical significance.

Pielou's evenness among gut region (LME results)

Species	Gut Region	Small Intestine Est.	Small Intestine SE	Small Intestine *p*	Cecum Est.	Cecum SE	Cecum *p*	Colon Est.	Colon SE	Colon *p*	Rectum Est.	Rectum SE	Rectum *p*
A. speciosus	Small intestine	–	–	–	-0.512	0.037	< **.001**	-0.461	0.033	< **.001**	-0.487	0.038	< **.001**
	Cecum	0.512	0.037	< **.001**	–	–	–	0.051	0.038	0.178	0.025	0.042	0.556
	Colon	0.461	0.033	< **.001**	-0.051	0.038	0.178	–	–	–	-0.026	0.039	0.498
	Rectum	0.487	0.038	< **.001**	-0.025	0.042	0.556	0.026	0.039	0.498	–	–	–
	Sex (male)	0.07	0.027	**0.011**	0.07	0.027	**0.011**	0.07	0.027	**0.011**	0.07	0.027	**0.011**
	Age	0.043	0.032	0.191	0.043	0.032	0.191	0.043	0.032	0.191	0.043	0.032	0.191
A. argenteus	Small intestine	–	–	–	-0.698	0.078	< **.001**	-0.694	0.083	< **.001**	-0.7	0.078	< **.001**
	Cecum	0.698	0.078	< **.001**	–	–	–	0.005	0.083	0.954	-0.001	0.078	0.983
	Colon	0.694	0.083	< **.001**	-0.005	0.083	0.954	–	–	–	-0.007	0.083	0.938
	Rectum	0.7	0.078	< **.001**	0.002	0.078	0.983	0.007	0.083	0.938	–	–	–
	Sex (male)	-0.199	0.057	**0.001**	-0.199	0.057	**0.002**	-0.199	0.057	**0.002**	-0.199	0.057	**0.002**
	Age	–	–	–	–	–	–	–	–	–	–	–	–
M. rufocanus	Small intestine	–	–	–	-0.259	0.051	< **.001**	-0.252	0.036	< **.001**	-0.273	0.049	< **.001**
	Cecum	0.259	0.051	< **.001**	–	–	–	0.007	0.051	0.895	-0.014	0.06	0.814
	Colon	0.252	0.036	< **.001**	-0.007	0.051	0.895	–	–	–	-0.021	0.049	0.675
	Rectum	0.273	0.049	< **.001**	0.014	0.06	0.814	0.021	0.049	0.675	–	–	–
	Sex (male)	0.038	0.032	0.24	0.038	0.032	0.24	0.038	0.032	0.24	0.038	0.032	0.24
	Age	-0.024	0.038	0.538	-0.024	0.038	0.536	-0.024	0.038	0.536	-0.024	0.038	0.536

Table S6: Linear mixed effects model results for Pielou's evenness comparing gut region within each species, values rounded to the thousandth place. Column is the response variable, rows are explanatory variables. Bold text indicates statistical significance.

Number of ASVs among gut region (LME results)

Species	Gut Region	Small Intestine Est.	Small Intestine SE	Small Intestine *p*	Cecum Est.	Cecum SE	Cecum *p*	Colon Est.	Colon SE	Colon *p*	Rectum Est.	Rectum SE	Rectum *p*
A. speciosus	Small intestine	–	–	–	-1.056	0.081	< **.001**	-1.107	0.071	< **.001**	-1.108	0.083	< **.001**
	Cecum	1.056	0.081	< **.001**	–	–	–	-0.051	0.081	0.531	-0.051	0.09	0.572
	Colon	1.107	0.071	< **.001**	0.051	0.081	0.531	–	–	–	-0.0002	0.083	0.998
	Rectum	1.108	0.083	< **.001**	0.051	0.09	0.572	0.0002	0.083	0.998	–	–	–
	Sex (male)	0.3	0.059	< **.001**	0.3	0.059	< **.001**	0.3	0.059	< **.001**	0.3	0.059	< **.001**
	Age	0.120	0.07	0.087	0.12	0.07	0.087	0.12	0.07	0.087	0.12	0.07	0.087
A. argenteus	Small intestine	–	–	–	-1.408	0.174	< **.001**	-1.169	0.187	< **.001**	-1.354	0.174	< **.001**
	Cecum	1.408	0.174	< **.001**	–	–	–	0.239	0.187	0.211	0.054	0.174	0.761
	Colon	1.169	0.187	< **.001**	-0.239	0.187	0.211	–	–	–	-0.185	0.187	0.33
	Rectum	1.354	0.174	< **.001**	-0.054	0.174	0.761	0.185	0.187	0.33	–	–	–
	Sex (male)	-0.432	0.128	**0.002**	-0.432	0.128	**0.002**	-0.432	0.128	**0.002**	-0.432	0.128	**0.002**
	Age	–	–	–	–	–	–	–	–	–	–	–	–
M. rufocanus	Small intestine	–	–	–	-1.028	0.129	< **.001**	-0.905	0.092	< **.001**	-0.92	0.124	< **.001**
	Cecum	1.028	0.129	< **.001**	–	–	–	0.123	0.129	0.343	0.108	0.151	0.478
	Colon	0.905	0.092	< **.001**	-0.123	0.129	0.343	–	–	–	-0.015	0.125	0.904
	Rectum	0.920	0.124	< **.001**	-0.108	0.151	0.478	0.015	0.125	0.904	–	–	–
	Sex (male)	-0.043	0.082	0.6	-0.043	0.082	0.6	-0.043	0.082	0.6	-0.043	0.082	0.6
	Age	-0.11	0.097	0.261	-0.11	0.097	0.261	-0.11	0.097	0.261	-0.11	0.097	0.261

Table S7: Linear mixed effects model results for the number of ASVs comparing gut region within each species, values rounded to the thousandth place. Column is the response variable, rows are explanatory variables. Bold text indicates statistical significance.

Shannon's diversity among species (LME results)

Gut Region	Species	*A. speciosus*			*A. argenteus*			*M. rufocanus*		
		**Est.**	**SE**	***p***	**Est.**	**SE**	***p***	**Est.**	**SE**	***p***
**Rectum**	*A. speciosus*	–	–	–	0.064	0.036	0.079	-0.165	0.035	< **.001**
	A. argenteus	-0.064	0.036	0.079	–	–	–	-0.229	0.047	< **.001**
	M. rufocanus	0.165	0.035	< **.001**	0.229	0.047	< **.001**	–	–	–
	Sex (male)	0.004	0.026	0.877	0.004	0.026	0.877	0.004	0.026	0.877
	Age	0.019	0.037	0.618	0.019	0.037	0.618	0.019	0.037	0.618
**Colon**	*A. speciosus*	–	–	–	0.084	0.04	**0.039**	-0.174	0.025	< **.001**
	A. argenteus	-0.084	0.04	**0.039**	–	–	–	-0.259	0.045	< **.001**
	M. rufocanus	0.174	0.025	< **.001**	0.259	0.045	< **.001**	–	–	–
	Sex (male)	-0.001	0.021	0.981	-0.001	0.021	0.981	-5E-04	0.021	0.981
	Age	0.015	0.024	0.554	0.014	0.024	0.554	0.014	0.024	0.554
**Cecum**	*A. speciosus*	–	–	–	0.079	0.029	**0.009**	-0.15	0.03	< **.001**
	A. argenteus	-0.079	0.029	**0.009**	–	–	–	-0.228	0.039	< **.001**
	M. rufocanus	0.15	0.03	< **.001**	0.228	0.039	< **.001**	–	–	–
	Sex (male)	0.014	0.021	0.507	0.013	0.021	0.507	0.014	0.021	0.507
	Age	-0.002	0.03	0.955	-0.002	0.03	0.955	-0.002	0.03	0.955
**Small Intestine**	*A. speciosus*	–	–	–	0.372	0.138	**0.009**	-0.476	0.092	< **.001**
	A. argenteus	-0.373	0.138	**0.009**	–	–	–	-0.849	0.153	< **.001**
	M. rufocanus	0.476	0.092	< **.001**	0.849	0.153	< **.001**	–	–	–
	Sex (male)	0.07	0.077	0.368	0.07	0.077	0.368	0.07	0.077	0.368
	Age	-0.048	0.092	0.604	-0.048	0.092	0.604	-0.048	0.092	0.604

Table S8: Linear mixed effects model results for Shannon diversity comparing each gut region among species, values rounded to the thousandth place. Column is the response variable, rows are explanatory variables. Bold text indicates statistical significance.

Faith's PD among species (LME results)

Gut Region	Species	*A. speciosus* Est.	*A. speciosus* SE	*A. speciosus* *p*	*A. argenteus* Est.	*A. argenteus* SE	*A. argenteus* *p*	*M. rufocanus* Est.	*M. rufocanus* SE	*M. rufocanus* *p*
**Rectum**	*A. speciosus*	–	–	–	0.053	0.036	0.152	-0.143	0.035	**<.001**
	A. argenteus	-0.053	0.036	0.152	–	–	–	-0.196	0.046	**< .001**
	M. rufocanus	0.143	0.035	**< .001**	0.196	0.046	**< .001**	–	–	–
	Sex (male)	-0.022	0.027	0.418	-0.022	0.027	0.418	-0.022	0.027	0.418
	Age	-0.044	0.036	0.234	-0.044	0.036	0.234	-0.044	0.036	0.234
**Colon**	*A. speciosus*	–	–	–	0.147	0.046	**0.002**	-0.096	0.029	**0.001**
	A. argenteus	-0.147	0.046	**0.002**	–	–	–	-0.242	0.051	**< .001**
	M. rufocanus	0.096	0.029	**0.001**	0.242	0.051	**< .001**	–	–	–
	Sex (male)	0.011	0.024	0.643	0.011	0.024	0.643	0.011	0.024	0.643
	Age	-0.008	0.028	0.763	-0.008	0.028	0.763	-0.008	0.028	0.763
**Cecum**	*A. speciosus*	–	–	–	0.014	0.037	0.712	-0.228	0.038	**< .001**
	A. argenteus	-0.014	0.037	0.712	–	–	–	-0.242	0.05	**< .001**
	M. rufocanus	0.228	0.038	**< .001**	0.242	0.05	**< .001**	–	–	–
	Sex (male)	0.003	0.027	0.906	0.003	0.027	0.906	0.003	0.027	0.906
	Age	-0.054	0.039	0.176	-0.054	0.039	0.176	-0.054	0.039	0.176
**Small Intestine**	*A. speciosus*	–	–	–	0.088	0.124	0.478	-0.057	0.095	0.55
	A. argenteus	-0.088	0.124	0.478	–	–	–	-0.146	0.154	0.346
	M. rufocanus	0.057	0.095	0.55	0.146	0.154	0.346	–	–	–
	Sex (male)	0.062	0.067	0.361	0.062	0.067	0.361	0.062	0.067	0.361
	Age	-0.072	0.083	0.383	-0.072	0.083	0.383	-0.072	0.083	0.383

Table S9: Linear mixed effects model results for Faith's phylogenetic diversity comparing each gut region among species, values rounded to the thousandth place. Column is the response variable, rows are explanatory variables. Bold text indicates statistical significance.

Pielou's evenness among species (LME results)

Gut Region	Species	*A. speciosus* Est.	*A. speciosus* SE	*A. speciosus* *p*	*A. argenteus* Est.	*A. argenteus* SE	*A. argenteus* *p*	*M. rufocanus* Est.	*M. rufocanus* SE	*M. rufocanus* *p*
**Rectum**	*A. speciosus*	–	–	–	0.046	0.025	0.073	-0.099	0.025	< **.001**
	A. argenteus	-0.046	0.025	0.073	–	–	–	-0.145	0.033	< **.001**
	M. rufocanus	0.1	0.025	< **.001**	0.145	0.033	< **.001**	–	–	–
	Sex (male)	-0.003	0.018	0.858	-0.003	0.018	0.858	-0.003	0.018	0.858
	Age	0.02	0.026	0.459	0.02	0.026	0.459	0.02	0.026	0.459
**Colon**	*A. speciosus*	–	–	–	0.028	0.029	0.339	-0.109	0.019	< **.001**
	A. argenteus	-0.028	0.029	0.339	–	–	–	-0.137	0.033	< **.001**
	M. rufocanus	0.109	0.019	< **.001**	0.137	0.033	< **.001**	–	–	–
	Sex (male)	-0.004	0.015	0.803	-0.004	0.015	0.803	-0.004	0.015	0.803
	Age	0.015	0.018	0.412	0.015	0.018	0.412	0.015	0.018	0.412
**Cecum**	*A. speciosus*	–	–	–	0.078	0.019	< **.001**	-0.054	0.02	**0.009**
	A. argenteus	-0.078	0.019	< **.001**	–	–	–	-0.132	0.026	< **.001**
	M. rufocanus	0.054	0.02	**0.009**	0.132	0.026	< **.001**	–	–	–
	Sex (male)	0.003	0.013	0.83	0.003	0.013	0.83	0.003	0.013	0.83
	Age	0.012	0.02	0.563	0.012	0.02	0.563	0.012	0.02	0.563
**Small Intestine**	*A. speciosus*	–	–	–	0.273	0.1	**0.008**	-0.341	0.066	< **.001**
	A. argenteus	-0.273	0.1	**0.008**	–	–	–	-0.614	0.11	< **.001**
	M. rufocanus	0.341	0.066	< **.001**	0.614	0.11	< **.001**	–	–	–
	Sex (male)	0.055	0.056	0.327	0.055	0.056	0.327	0.055	0.056	0.327
	Age	-0.018	0.066	0.785	-0.018	0.066	0.785	-0.018	0.066	0.785

Table S10: Linear mixed effects model results for Pielou's evenness comparing each gut region among species, values rounded to the thousandth place. Column is the response variable, rows are explanatory variables. Bold text indicates statistical significance.

Number of ASVs among species (LME results)

Gut Region	Species	A. speciosus Est.	A. speciosus SE	A. speciosus *p*	*A. argenteus* Est.	*A. argenteus* SE	*A. argenteus* *p*	*M. rufocanus* Est.	*M. rufocanus* SE	*M. rufocanus* *p*
**Rectum**	*A. speciosus*	–	–	–	0.1	0.074	0.188	-0.377	0.072	< **.001**
	A. argenteus	-0.1	0.074	0.188	–	–	–	-0.477	0.094	< **.001**
	M. rufocanus	0.377	0.072	< **.001**	0.477	0.094	< **.001**	–	–	–
	Sex (male)	0.037	0.055	0.499	0.037	0.055	0.499	0.037	0.055	0.499
	Age	0.002	0.074	0.977	0.002	0.074	0.977	0.002	0.074	0.977
**Colon**	*A. speciosus*	–	–	–	0.297	0.084	< **.001**	-0.369	0.051	< **.001**
	A. argenteus	-0.297	0.084	< **.001**	–	–	–	-0.667	0.092	< **.001**
	M. rufocanus	0.369	0.051	< **.001**	0.667	0.092	< **.001**	–	–	–
	Sex (male)	0.019	0.044	0.66	0.019	0.043	0.66	0.019	0.044	0.66
	Age	-0.002	0.051	0.962	-0.002	0.051	0.962	-0.002	0.051	0.962
**Cecum**	*A. speciosus*	–	–	–	0.008	0.089	0.93	-0.529	0.091	< **.001**
	A. argenteus	-0.008	0.089	0.93	–	–	–	-0.537	0.117	< **.001**
	M. rufocanus	0.529	0.091	< **.001**	0.537	0.117	< **.001**	–	–	–
	Sex (male)	0.062	0.065	0.345	0.062	0.065	0.345	0.062	0.065	0.345
	Age	-0.039	0.091	0.668	-0.039	0.091	0.668	-0.039	0.091	0.668
**Small Intestine**	*A. speciosus*	–	–	–	0.399	0.229	0.085	-0.634	0.154	< **.001**
	A. argenteus	-0.399	0.229	0.085	–	–	–	-1.033	0.255	< **.001**
	M. rufocanus	0.634	0.154	< **.001**	1.033	0.255	< **.001**	–	–	–
	Sex (male)	0.069	0.128	0.589	0.069	0.128	0.589	0.069	0.128	0.589
	Age	-0.131	0.152	0.391	-0.131	0.152	0.391	-0.131	0.152	0.391

Table S11: Linear mixed effects model results for the number of ASVs comparing each gut region among species, values rounded to the thousandth place. Column is the response variable, rows are explanatory variables. Bold text indicates statistical significance.

PERMANOVA all gut regions included, *A. speciosus*

Dissimilarity Index	Variable	Sum of Squares	R^2	F	*p*
Jaccard	Gut Region	5.021	0.10766	5.7211	**0.001**
	Site	3.309	0.07096	3.7708	**0.001**
	Sex	0.799	0.01713	2.7304	**0.001**
	Age	0.647	0.01388	2.2126	**0.002**
Bray-Curtis	Gut Region	8.511	0.17782	10.3656	**0.001**
	Site	3.272	0.06837	3.9852	**0.001**
	Sex	0.793	0.01658	2.8988	**0.002**
	Age	0.8	0.01672	2.9242	**0.002**
Unweighted unifrac	Gut Region	3.1658	0.20183	12.2717	**0.001**
	Site	1.167	0.0744	4.5235	**0.001**
	Sex	0.2774	0.01769	3.2259	**0.004**
	Age	0.2402	0.01531	2.7934	**0.003**
Weighted unifrac	Gut Region	5.6009	0.342	24.7671	**0.001**
	Site	0.7453	0.04551	3.2956	**0.002**
	Sex	0.1828	0.01116	2.4248	0.055
	Age	0.3501	0.02138	4.644	**0.002**

Table S12: PERMANOVA results for within species (*A. speciosus)* gut microbiota beta-diversity with all GIT regions included. Bold text indicates statistical significance.

PERMANOVA gut regions included, *A. argenteus*

Dissimilarity Index	Variable	Sum of Squares	R^2	F	*p*
Jaccard	Gut Region	1.737	0.15734	2.0728	**0.001**
	Site	0.8825	0.07994	3.1595	**0.001**
	Sex	0.5989	0.05425	2.1442	**0.002**
Bray-Curtis	Gut Region	2.3742	0.20174	2.8253	**0.001**
	Site	0.8956	0.0761	3.1972	**0.001**
	Sex	0.6556	0.0557	2.3403	**0.006**
Unweighted unifrac	Gut Region	1.0521	0.29497	4.6851	**0.001**
	Site	0.2206	0.06186	2.9473	**0.006**
	Sex	0.1981	0.05554	2.6464	**0.014**
Weighted unifrac	Gut Region	3.1108	0.47008	10.0174	**0.001**
	Site	0.4098	0.06193	3.9593	**0.014**
	Sex	0.1986	0.03001	1.9183	0.115

Table S13: PERMANOVA results for within species (*A. argenteus)* gut microbiota beta-diversity with all GIT regions included. Bold text indicates statistical significance.

PERMANOVA gut regions included, *M. rufocanus*

Dissimilarity Index	Variable	Sum of Squares	R^2	F	*p*
Jaccard	Gut Region	3.5	0.08417	3.5317	**0.001**
	Site	4.086	0.09826	4.1229	**0.001**
	Sex	0.621	0.01493	1.8801	**0.003**
	Age	0.674	0.0162	2.0394	**0.001**
Bray-Curtis	Gut Region	4.629	0.11182	5.0003	**0.001**
	Site	4.697	0.11345	5.0732	**0.001**
	Sex	0.646	0.01561	2.0943	**0.001**
	Age	0.874	0.02112	2.8334	**0.001**
Unweighted unifrac	Gut Region	3.2729	0.2294	11.5249	**0.001**
	Site	1.2335	0.08646	4.3436	**0.001**
	Sex	0.1512	0.0106	1.5972	0.071
	Age	0.2382	0.0167	2.5167	**0.007**
Weighted unifrac	Gut Region	3.5355	0.25814	12.9768	**0.001**
	Site	0.6179	0.04511	2.2678	**0.026**
	Sex	0.1099	0.00802	1.2096	0.266
	Age	0.4423	0.03229	4.8702	**0.006**

Table S14: PERMANOVA results for within species (*M. rufocanus)* gut microbiota beta-diversity with all GIT regions included. Bold text indicates statistical significance.

Pairwise PERMANOVA among gut regions, *A. speciosus*

Dissimilarity Index	Variable	Cecum - Rectum				Colon - Cecum				Colon - Rectum			
		**Sum of Squares**	**R^2**	**F**	***p***	**Sum of Squares**	**R^2**	**F**	***p***	**Sum of Squares**	**R^2**	**F**	***p***
Jaccard	Gut Region	0.1699	0.01125	0.6435	0.983	0.2749	0.01376	1.0176	0.413	0.3165	0.01615	1.1588	0.129
	Site	2.0954	0.13866	2.6447	**0.001**	2.2219	0.11127	2.7421	**0.001**	2.1915	0.11184	2.6743	**0.001**
	Sex	0.5117	0.03386	1.9373	**0.001**	0.5082	0.02545	1.8815	**0.001**	0.4807	0.02453	1.7599	**0.003**
	Age	0.4507	0.02982	1.7063	**0.005**	0.4883	0.02445	1.808	**0.001**	0.4903	0.02502	1.7948	**0.002**
Bray-Curtis	Gut Region	0.4834	0.03205	1.879	**0.005**	0.5686	0.02823	2.144	**0.001**	0.4786	0.02377	1.7421	**0.009**
	Site	1.8524	0.1228	2.4001	**0.001**	2.1865	0.10855	2.7482	**0.001**	2.2107	0.10981	2.6826	**0.001**
	Sex	0.5169	0.03427	2.0093	**0.005**	0.5016	0.0249	1.8913	**0.005**	0.5203	0.02584	1.8939	**0.006**
	Age	0.655	0.04342	2.546	**0.002**	0.7085	0.03517	2.6713	**0.001**	0.7159	0.03556	2.6062	**0.001**
Unweighted unifrac	Gut Region	0.0546	0.01486	0.8597	0.68	0.0672	0.01415	1.0655	0.357	0.0881	0.01776	1.2976	0.102
	Site	0.4848	0.13187	2.5424	**0.001**	0.5333	0.11224	2.8184	**0.001**	0.5635	0.1136	2.7671	**0.001**
	Sex	0.1265	0.03441	1.9904	**0.007**	0.1377	0.02898	2.1833	**0.003**	0.1387	0.02796	2.043	**0.002**
	Age	0.15	0.0408	2.3599	**0.003**	0.1656	0.03485	2.6254	**0.002**	0.165	0.03326	2.4305	**0.002**
Weighted unifrac	Gut Region	0.1996	0.05713	3.6447	**0.011**	0.1469	0.03054	2.4485	**0.04**	0.2602	0.05145	4.1045	**0.006**
	Site	0.3712	0.10626	2.2596	**0.018**	0.3991	0.08295	2.217	**0.013**	0.465	0.09195	2.4452	**0.005**
	Sex	0.1282	0.03671	2.342	**0.047**	0.1318	0.02739	2.196	0.059	0.1691	0.03344	2.6678	**0.026**
	Age	0.3302	0.09452	6.0301	**0.001**	0.4732	0.09834	7.8853	**0.001**	0.4229	0.08362	6.6707	**0.001**

Dissimilarity Index	Variable	Colon - Small Intestine				Small Intestine - Cecum				Small Intestine - Rectum			
		**Sum of Squares**	**R^2**	**F**	**p**	**Sum of Squares**	**R^2**	**F**	**p**	**Sum of Squares**	**R^2**	**F**	**p**
Jaccard	Gut Region	3.2263	0.10765	10.2769	**0.001**	2.7766	0.11057	8.7412	**0.001**	2.4068	0.09892	7.4833	**0.001**
	Site	1.881	0.06276	1.9972	**0.001**	1.741	0.06933	1.827	**0.001**	1.7266	0.07096	1.7894	**0.001**
	Sex	0.5181	0.01729	1.6504	**0.009**	0.528	0.02102	1.6622	**0.01**	0.5192	0.02134	1.6143	**0.008**
	Age	0.4845	0.01617	1.5434	**0.026**	0.3723	0.01482	1.172	0.139	0.3804	0.01564	1.1828	0.114
Bray-Curtis	Gut Region	5.2863	0.17607	18.4936	**0.001**	4.9493	0.20059	17.7443	**0.001**	4.1016	0.17185	14.3382	**0.001**
	Site	2.0776	0.0692	2.4227	**0.001**	1.6124	0.06535	1.9269	**0.001**	1.7401	0.07291	2.0277	**0.003**
	Sex	0.4588	0.01528	1.6052	0.068	0.4413	0.01789	1.5823	0.072	0.4688	0.01964	1.6388	0.058
	Age	0.4773	0.0159	1.6698	**0.05**	0.3769	0.01528	1.3513	0.139	0.3925	0.01645	1.372	0.125
Unweighted unifrac	Gut Region	2.1938	0.20393	22.9577	**0.001**	1.7389	0.19544	17.7268	**0.001**	1.5892	0.18596	16.0504	**0.001**
	Site	0.8773	0.08155	3.0601	**0.001**	0.7339	0.08248	2.4938	**0.002**	0.6843	0.08007	2.3037	**0.001**
	Sex	0.2519	0.02341	2.6359	**0.014**	0.2304	0.02589	2.3483	**0.023**	0.2257	0.02641	2.2796	**0.024**
	Age	0.1724	0.01602	1.8041	**0.07**	0.1124	0.01264	1.1462	0.293	0.1057	0.01237	1.0678	0.357
Weighted unifrac	Gut Region	3.2353	0.30236	37.7693	**0.001**	3.2278	0.35068	38.4697	**0.001**	3.455	0.36075	39.0967	**0.001**
	Site	0.6223	0.05816	2.4216	**0.007**	0.4872	0.05293	1.9354	**0.033**	0.5295	0.05528	1.9972	**0.038**
	Sex	0.1497	0.01399	1.748	0.123	0.1982	0.02154	2.3627	0.066	0.2001	0.02089	2.2638	0.059
	Age	0.1825	0.01706	2.131	0.077	0.089	0.00967	1.061	0.327	0.0905	0.00945	1.0242	0.373

Table S15: Pairwise PERMANOVA results for within species (*A. speciosus*) gut microbiota beta-diversity. Bold text indicates statistical significance.

Pairwise PERMANOVA among gut regions, *A. argenteus*

Dissimilarity Index	Variable	Cecum - Rectum				Colon - Cecum				Colon - Rectum			
		**Sum of Squares**	**R^2**	**F**	***p***	**Sum of Squares**	**R^2**	**F**	***p***	**Sum of Squares**	**R^2**	**F**	***p***
Jaccard	Gut Region	0.0922	0.02049	0.3875	0.986	0.1474	0.03588	0.5968	0.894	0.1114	0.02683	0.4397	0.984
	Site	0.6567	0.14596	2.7608	**0.001**	0.5944	0.14466	2.4061	**0.001**	0.603	0.14518	2.3791	**0.001**
	Sex	0.4203	0.09341	1.7668	**0.01**	0.4028	0.09802	1.6304	**0.022**	0.3977	0.09574	1.5689	**0.04**
Bray-Curtis	Gut Region	0.1461	0.0308	0.5764	0.878	0.1406	0.03317	0.5432	0.912	0.131	0.0293	0.4851	0.947
	Site	0.6613	0.13944	2.6097	**0.004**	0.5645	0.13314	2.1808	**0.008**	0.6637	0.14842	2.4572	**0.001**
	Sex	0.3875	0.0817	1.5289	0.094	0.4285	0.10107	1.6555	0.061	0.4357	0.09744	1.6131	0.059
Unweighted unifrac	Gut Region	0.02864	0.02698	0.5118	0.936	0.0444	0.046	0.7767	0.709	0.02653	0.02806	0.4579	0.973
	Site	0.15127	0.14254	2.7037	**0.003**	0.13992	0.14495	2.4473	**0.003**	0.1376	0.14552	2.3749	**0.001**
	Sex	0.09809	0.09243	1.7533	0.035	0.09491	0.09833	1.6602	0.055	0.08618	0.09114	1.4875	0.11
Weighted unifrac	Gut Region	0.1019	0.07118	1.5253	0.172	0.08783	0.05638	1.0206	0.387	0.10634	0.06438	1.3562	0.213
	Site	0.32197	0.22491	4.8194	**0.001**	0.31455	0.2019	3.655	**0.007**	0.4726	0.28612	6.0277	**0.002**
	Sex	0.07241	0.05058	1.0839	0.364	0.12286	0.07886	1.4276	0.199	0.13194	0.07988	1.6828	0.141

Dissimilarity Index	Variable	Colon - Small Intestine				Small Intestine - Cecum				Small Intestine - Rectum			
		Sum of Squares	**R^2**	F	p	Sum of Squares	**R^2**	F	p	Sum of Squares	**R^2**	F	p
Jaccard	Gut Region	0.9049	0.15698	2.7296	**0.001**	1.1474	0.17836	3.6504	**0.001**	1.0643	0.16777	3.4144	**0.001**
	Site	0.4479	0.07769	1.351	0.072	0.4819	0.07491	1.5331	**0.049**	0.4847	0.0764	1.5548	**0.039**
	Sex	0.4336	0.07522	1.3079	0.103	0.4032	0.06269	1.283	0.121	0.4309	0.06793	1.3824	0.078
Bray-Curtis	Gut Region	1.277	0.21026	3.9726	**0.001**	1.5953	0.24005	5.283	**0.001**	1.3492	0.20287	4.3108	**0.001**
	Site	0.5194	0.08552	1.6157	0.058	0.4569	0.06875	1.513	0.087	0.4976	0.07482	1.5897	**0.07**
	Sex	0.4196	0.06909	1.3053	0.169	0.3658	0.05505	1.2115	0.237	0.4219	0.06344	1.348	0.156
Unweighted unifrac	Gut Region	0.56323	0.28141	5.838	**0.001**	0.75253	0.32092	8.177	**0.001**	0.74469	0.32155	8.4404	**0.001**
	Site	0.09295	0.04644	0.9635	0.385	0.12238	0.05219	1.3298	0.208	0.14274	0.06163	1.6178	0.104
	Sex	0.18757	0.09372	1.9442	**0.049**	0.18161	0.07745	1.9734	0.063	0.19331	0.08347	2.191	**0.046**
Weighted unifrac	Gut Region	1.5613	0.41869	10.6232	**0.001**	1.9941	0.50749	16.7913	**0.001**	2.225	0.52283	18.3957	**0.001**
	Site	0.2119	0.05683	1.442	0.191	0.1108	0.0282	0.9331	0.379	0.1655	0.0389	1.3686	0.225
	Sex	0.1921	0.05153	1.3073	0.237	0.1618	0.04118	1.3626	0.226	0.1718	0.04038	1.4207	0.208

Table S16: Pairwise PERMANOVA results for within species (*A. argenteus)* gut microbiota beta-diversity. Bold text indicates statistical significance.

Pairwise PERMANOVA among gut regions, *M. rufocanus*

Dissimilarity Index	Variable	Cecum - Rectum				Colon - Cecum				Colon - Rectum			
		**Sum of Squares**	**R^2**	**F**	***p***	**Sum of Squares**	**R^2**	**F**	***p***	**Sum of Squares**	**R^2**	**F**	***p***
Jaccard	Gut Region	0.1941	0.01999	0.707	0.916	0.2469	0.01409	0.8036	0.928	0.2572	0.01419	0.8193	0.912
	Site	2.4425	0.2515	2.9648	**0.001**	2.5426	0.14508	2.7583	**0.001**	2.5503	0.14069	2.7078	**0.001**
	Sex	0.531	0.05467	1.9335	**0.004**	0.4544	0.02593	1.4788	**0.015**	0.4456	0.02458	1.4194	**0.019**
	Age	0.5027	0.05176	1.8307	**0.002**	0.4545	0.02594	1.4793	**0.011**	0.4321	0.02384	1.3765	**0.026**
Bray-Curtis	Gut Region	0.1664	0.01804	0.7026	0.873	0.2305	0.01362	0.8015	0.856	0.267	0.01527	0.9144	0.643
	Site	2.7607	0.29933	3.885	**0.001**	2.8832	0.17041	3.3419	**0.001**	2.9234	0.16723	3.3377	**0.001**
	Sex	0.5524	0.0599	2.3324	**0.002**	0.4099	0.02423	1.4253	**0.031**	0.4159	0.02379	1.4244	**0.04**
	Age	0.5322	0.0577	2.2468	**0.003**	0.4547	0.02688	1.5813	**0.022**	0.4452	0.02547	1.525	**0.022**
Unweighted unifrac	Gut Region	0.06482	0.02881	1.119	0.292	0.0879	0.02213	1.3233	0.096	0.0666	0.01614	0.9697	0.476
	Site	0.68059	0.30254	3.9162	**0.001**	0.6834	0.17202	3.4292	**0.001**	0.6886	0.16698	3.3437	**0.001**
	Sex	0.11133	0.04949	1.9219	**0.008**	0.0872	0.02196	1.3132	0.099	0.091	0.02206	1.3254	0.078
	Age	0.11839	0.05263	2.0437	**0.003**	0.125	0.03148	1.8825	**0.009**	0.12	0.02909	1.7478	**0.013**
Weighted unifrac	Gut Region	0.04721	0.04381	1.7634	0.089	0.08447	0.03909	2.2624	**0.041**	0.0878	0.03967	2.3492	**0.041**
	Site	0.26678	0.24756	3.3216	**0.001**	0.23425	0.10841	2.0913	**0.004**	0.22869	0.10334	2.0397	**0.014**
	Sex	0.08093	0.0751	3.023	**0.004**	0.05941	0.02749	1.5911	0.112	0.07802	0.03526	2.0876	0.063
	Age	0.09371	0.08696	3.5004	**0.003**	0.10243	0.0474	2.7433	**0.015**	0.09925	0.04485	2.6557	**0.019**

Dissimilarity Index	Variable	Colon - Small Intestine				Small Intestine - Cecum				Small Intestine - Rectum			
		**Sum of Squares**	**R^2**	**F**	**p**	**Sum of Squares**	**R^2**	**F**	**p**	**Sum of Squares**	**R^2**	**F**	**p**
Jaccard	Gut Region	2.3792	0.0768	6.8205	**0.001**	1.4535	0.06693	4.0903	**0.001**	1.3684	0.0616	3.8015	**0.001**
	Site	2.563	0.08274	2.4492	**0.001**	2.3521	0.10832	2.2064	**0.001**	2.3327	0.10501	2.1601	**0.001**
	Sex	0.4127	0.01332	1.1831	0.13	0.3657	0.01684	1.0291	0.347	0.3844	0.0173	1.0679	0.257
	Age	0.5075	0.01638	1.4548	**0.018**	0.4868	0.02242	1.3699	**0.027**	0.4903	0.02207	1.3619	**0.025**
Bray-Curtis	Gut Region	3.2344	0.10469	9.7927	**0.001**	1.9177	0.08953	5.7594	**0.001**	1.9786	0.08974	5.8709	**0.001**
	Site	2.7804	0.09	2.8061	**0.001**	2.4535	0.11455	2.4562	**0.001**	2.4501	0.11112	2.4233	**0.001**
	Sex	0.4024	0.01302	1.2182	0.143	0.3547	0.01656	1.0653	0.33	0.3737	0.01695	1.109	0.279
	Age	0.6967	0.02255	2.1095	**0.001**	0.7103	0.03316	2.1331	**0.002**	0.7319	0.03319	2.1715	**0.002**
Unweighted unifrac	Gut Region	2.4149	0.22128	23.4817	**0.001**	1.4157	0.17379	12.0178	**0.001**	1.3607	0.16447	11.2724	**0.001**
	Site	0.8109	0.0743	2.6283	**0.001**	0.803	0.09858	2.2722	**0.003**	0.7393	0.08936	2.0414	**0.002**
	Sex	0.1067	0.00978	1.038	0.318	0.0911	0.01119	0.7738	0.648	0.0882	0.01067	0.731	0.732
	Age	0.1761	0.01614	1.7125	0.062	0.1817	0.02231	1.5426	0.094	0.1701	0.02056	1.4092	0.144
Weighted unifrac	Gut Region	2.5543	0.21908	22.953	**0.001**	1.5439	0.16104	10.7244	**0.001**	1.5094	0.15706	10.6542	**0.001**
	Site	0.656	0.05626	1.9649	0.055	0.5964	0.06221	1.381	0.177	0.6114	0.06362	1.4385	0.159
	Sex	0.0712	0.00611	0.6398	0.596	0.0655	0.00683	0.4551	0.787	0.0704	0.00732	0.4969	0.756
	Age	0.3652	0.03132	3.2819	**0.029**	0.4708	0.04911	3.2702	**0.024**	0.4775	0.04968	3.3701	**0.023**

Table S17: Pairwise PERMANOVA results for within species (*M. rufocanus)* gut microbiota beta-diversity. Bold text indicates statistical significance.

PERMANOVA among host species, all species included

Dissimilarity Index	Variable	Rectum: Sum of squares	Rectum: R^2	Rectum: F	Rectum: *p*	Colon: Sum of squares	Colon: R^2	Colon: F	Colon: *p*
Jaccard	Species	4.1466	0.22154	6.7272	**0.001**	7.588	0.22193	12.1555	**0.001**
	Site	1.3082	0.0699	1.4149	**0.027**	1.593	0.0466	1.7016	**0.003**
	Sex	0.3179	0.01699	1.0316	0.287	0.312	0.00912	0.9994	0.324
	Age	0.3081	0.01646	0.9996	0.36	0.352	0.01031	1.1291	0.222
Bray-Curtis	Species	4.386	0.23187	7.1296	**0.001**	7.967	0.23028	12.8502	**0.001**
	Site	1.2794	0.06764	1.3865	**0.025**	1.718	0.04966	1.8474	**0.002**
	Sex	0.3038	0.01606	0.9877	0.411	0.318	0.0092	1.0268	0.349
	Age	0.3349	0.01771	1.0889	0.262	0.414	0.01198	1.3367	0.112
Unweighted unifrac	Species	1.811	0.3439	12.5438	**0.001**	3.4537	0.36371	24.7898	**0.001**
	Site	0.3408	0.06472	1.5738	**0.033**	0.4477	0.04714	2.1421	**0.005**
	Sex	0.084	0.01596	1.1641	0.238	0.0633	0.00666	0.9085	0.43
	Age	0.0706	0.01341	0.9779	0.355	0.0978	0.0103	1.4034	0.148
Weighted unifrac	Species	0.9121	0.23594	7.1953	**0.001**	1.5127	0.21585	11.5832	**0.001**
	Site	0.2055	0.05316	1.0807	0.336	0.256	0.03652	1.3066	0.156
	Sex	0.0851	0.022	1.342	0.192	0.0706	0.01007	1.0811	0.361
	Age	0.0645	0.01668	1.017	0.418	0.0756	0.01079	1.1578	0.286

Dissimilarity Index	Variable	Cecum: Sum of squares	Cecum: R^2	Cecum: F	Cecum: p	Small Intestine: Sum of squares	Small Intestine: R^2	Small Intestine: F	Small Intestine: p
Jaccard	Species	4.2995	0.2308	7.2481	**0.001**	6.148	0.1553	8.3689	**0.001**
	Site	1.2947	0.0695	1.4551	**0.013**	1.81	0.04572	1.6426	**0.001**
	Sex	0.2894	0.01553	0.9756	0.391	0.367	0.00927	0.9988	0.353
	Age	0.2879	0.01546	0.9708	0.354	0.41	0.01035	1.1158	0.199
Bray-Curtis	Species	4.9895	0.26674	8.7541	**0.001**	8.045	0.20842	11.9986	**0.001**
	Site	1.1855	0.06338	1.3867	**0.028**	1.582	0.04098	1.5728	**0.012**
	Sex	0.2238	0.01196	0.7852	0.796	0.335	0.00869	1.0001	0.43
	Age	0.3375	0.01804	1.1842	0.197	0.477	0.01235	1.4216	0.093
Unweighted unifrac	Species	1.8215	0.34797	13.0457	**0.001**	2.7611	0.17991	10.4283	**0.001**
	Site	0.3476	0.06641	1.6598	**0.023**	1.2569	0.0819	3.1646	**0.001**
	Sex	0.0605	0.01156	0.867	0.505	0.0839	0.00546	0.6335	0.83
	Age	0.0729	0.01392	1.0441	0.302	0.1249	0.00814	0.9434	0.489
Weighted unifrac	Species	0.8903	0.24793	7.7369	**0.001**	3.6992	0.21817	12.5477	**0.001**
	Site	0.1711	0.04765	0.9913	0.464	0.5485	0.03235	1.2403	0.251
	Sex	0.0297	0.00827	0.516	0.857	0.0864	0.0051	0.5864	0.695
	Age	0.0833	0.0232	1.4479	0.155	0.2398	0.01414	1.6266	0.14

Table S18: PERMANOVA results comparing gut regions among species, all species included. Bold text indicates statistical significance.

Pairwise PERMANOVA between *A. speciosus* and *M. rufocanus*

Dissimilarity Index	Variable	Rectum: Sum of squares	Rectum: R^2	Rectum: F	Rectum: *p*	Colon: Sum of squares	Colon: R^2	Colon: F	Colon: *p*
Jaccard	Species	3.1497	0.20371	10.0046	**0.001**	6.5373	0.20839	20.8669	**0.001**
	Site	1.3012	0.08416	1.3777	**0.036**	1.6034	0.05111	1.706	**0.002**
	Sex	0.3155	0.02041	1.0022	0.347	0.3157	0.01006	1.0076	0.349
	Age	0.3057	0.01977	0.9709	0.432	0.3575	0.0114	1.141	0.208
Bray-Curtis	Species	3.357	0.21663	10.8262	**0.001**	6.8858	0.21806	22.3311	**0.001**
	Site	1.2777	0.08246	1.3736	0.051	1.7597	0.05573	1.9023	**0.002**
	Sex	0.2964	0.01913	0.9558	0.451	0.3103	0.00983	1.0065	0.335
	Age	0.3324	0.02145	1.0719	0.261	0.421	0.01333	1.3654	0.112
Unweighted unifrac	Species	1.4584	0.33571	20.2105	**0.001**	2.9769	0.32936	39.5733	**0.001**
	Site	0.3535	0.08137	1.6328	**0.035**	0.4531	0.05013	2.0077	**0.012**
	Sex	0.0774	0.01783	1.0732	0.313	0.0789	0.00873	1.0489	0.295
	Age	0.0736	0.01695	1.0203	0.335	0.1134	0.01254	1.5068	0.115
Weighted unifrac	Species	0.69639	0.2371	12.1151	**0.001**	1.2944	0.21858	21.7946	**0.001**
	Site	0.23924	0.08146	1.3874	0.134	0.2423	0.04092	1.3599	0.14
	Sex	0.04199	0.0143	0.7305	0.595	0.0276	0.00466	0.4642	0.881
	Age	0.06257	0.0213	1.0886	0.348	0.0813	0.01373	1.3695	0.203

Dissimilarity Index	Variable	Cecum: Sum of squares	Cecum: R^2	Cecum: F	Cecum: p	Small Intestine: Sum of squares	Small Intestine: R^2	Small Intestine: F	Small Intestine: p
Jaccard	Species	3.3089	0.21426	10.9643	**0.001**	4.95	0.14003	13.5642	**0.001**
	Site	1.2758	0.08261	1.4092	**0.023**	1.84	0.05206	1.6809	**0.001**
	Sex	0.3062	0.01983	1.0145	0.335	0.415	0.01173	1.1363	0.175
	Age	0.2915	0.01888	0.9661	0.397	0.409	0.01158	1.1218	0.186
Bray-Curtis	Species	3.6814	0.24221	12.8325	**0.001**	6.535	0.19063	19.7864	**0.001**
	Site	1.1799	0.07763	1.3709	0.07	1.829	0.05336	1.8461	**0.002**
	Sex	0.2409	0.01585	0.8397	0.647	0.341	0.00995	1.0325	0.345
	Age	0.3433	0.02258	1.1966	0.178	0.474	0.01384	1.4365	0.093
Unweighted unifrac	Species	1.4552	0.33364	20.5476	**0.001**	2.3594	0.17321	18.5568	**0.001**
	Site	0.3451	0.07911	1.6241	**0.037**	1.3134	0.09643	3.4434	**0.001**
	Sex	0.0665	0.01526	0.9396	0.401	0.1645	0.01208	1.2937	0.202
	Age	0.0869	0.01992	1.2268	0.204	0.1209	0.00888	0.951	0.419
Weighted unifrac	Species	0.61768	0.23025	11.7861	**0.001**	2.5086	0.17325	17.2415	**0.001**
	Site	0.15268	0.05691	0.9711	0.491	0.6107	0.04218	1.3991	0.151
	Sex	0.03135	0.01169	0.5982	0.751	0.0762	0.00527	0.524	0.758
	Age	0.09912	0.03695	1.8913	0.076	0.226	0.01561	1.5531	0.153

Table S19: Pairwise PERMANOVA results comparing gut regions between *A. speciosus* and *M. rufocanus*. Bold text indicates statistical significance.

Pairwise PERMANOVA between *A. speciosus* and *A. argenteus*

Dissimilarity Index	Variable	Rectum: Sum of squares	Rectum: R^2	Rectum: F	Rectum: *p*	Colon: Sum of squares	Colon: R^2	Colon: F	Colon: *p*
Jaccard	Species	0.7782	0.07587	2.7271	**0.001**	0.7655	0.05248	2.661	**0.001**
	Site	1.1868	0.11571	1.3864	**0.001**	1.3609	0.09331	1.5769	**0.001**
	Sex	0.2901	0.02828	1.0166	0.381	0.3077	0.0211	1.0698	0.216
	Age	0.2974	0.02899	1.0422	0.329	0.3568	0.02446	1.2402	**0.042**
Bray-Curtis	Species	0.816	0.07714	2.735	**0.001**	0.8344	0.05535	2.8639	**0.001**
	Site	1.0608	0.10028	1.1851	0.095	1.4731	0.09772	1.6854	**0.001**
	Sex	0.282	0.02666	0.9451	0.574	0.3313	0.02198	1.1373	0.245
	Age	0.3632	0.03433	1.2172	0.153	0.49	0.0325	1.6817	**0.014**
Unweighted unifrac	Species	0.18585	0.07569	2.7162	**0.001**	0.2388	0.06516	3.4343	**0.001**
	Site	0.26102	0.1063	1.2716	**0.027**	0.3698	0.10091	1.7728	**0.001**
	Sex	0.07048	0.0287	1.0301	0.418	0.0796	0.02172	1.145	0.2
	Age	0.09076	0.03696	1.3264	0.075	0.1257	0.03431	1.8081	**0.005**
Weighted unifrac	Species	0.10315	0.04071	1.4155	0.194	0.1422	0.03478	1.8977	0.098
	Site	0.22207	0.08765	1.0158	0.422	0.3285	0.08039	1.4619	0.128
	Sex	0.13933	0.055	1.912	0.085	0.201	0.04917	2.6828	**0.028**
	Age	0.10138	0.04002	1.3913	0.182	0.344	0.08418	4.5928	**0.005**

Dissimilarity Index	Variable	Cecum: Sum of squares	Cecum: R^2	Cecum: F	Cecum: p	Small Intestine: Sum of squares	Small Intestine: R^2	Small Intestine: F	Small Intestine: p
Jaccard	Species	0.7785	0.0732	2.7953	**0.001**	0.7782	0.07587	2.7271	**0.001**
	Site	1.2264	0.11532	1.4678	**0.001**	1.1868	0.11571	1.3864	**0.001**
	Sex	0.2949	0.02773	1.059	0.275	0.2901	0.02828	1.0166	0.381
	Age	0.2581	0.02427	0.9268	0.687	0.2974	0.02899	1.0422	0.329
Bray-Curtis	Species	1.2237	0.11463	4.5261	**0.001**	0.816	0.07714	2.735	**0.001**
	Site	1.0294	0.09644	1.2692	**0.048**	1.0608	0.10028	1.1851	0.095
	Sex	0.2152	0.02016	0.7961	0.832	0.282	0.02666	0.9451	0.574
	Age	0.3658	0.03427	1.3531	0.099	0.3632	0.03433	1.2172	0.153
Unweighted unifrac	Species	0.20173	0.08141	3.1259	**0.001**	0.18585	0.07569	2.7162	**0.001**
	Site	0.2635	0.10634	1.361	**0.005**	0.26102	0.1063	1.2716	**0.027**
	Sex	0.07092	0.02862	1.0989	0.282	0.07048	0.0287	1.0301	0.418
	Age	0.0704	0.02841	1.0909	0.326	0.09076	0.03696	1.3264	0.075
Weighted unifrac	Species	0.23371	0.09595	3.7704	**0.003**	0.10315	0.04071	1.4155	0.194
	Site	0.20773	0.08529	1.1171	0.3	0.22207	0.08765	1.0158	0.422
	Sex	0.04129	0.01695	0.6661	0.68	0.13933	0.055	1.912	0.085
	Age	0.15533	0.06377	2.5058	**0.04**	0.10138	0.04002	1.3913	0.182

Table S20: Pairwise PERMANOVA results comparing gut regions between *A. speciosus* and *A. argenteus*. Bold text indicates statistical significance.

Pairwise PERMANOVA between *A. argenteus* and *M. rufocanus*

Dissimilarity Index	Variable	Rectum: Sum of squares	Rectum: R^2	Rectum: F	Rectum: *p*	Colon: Sum of squares	Colon: R^2	Colon: F	Colon: *p*
Jaccard	Species	2.134	0.22679	7.0295	**0.001**	2.1993	0.13112	6.7826	**0.001**
	Site	1.453	0.15442	1.5955	**0.009**	1.6035	0.0956	1.6484	**0.001**
	Sex	0.3226	0.03428	1.0625	0.275	0.3091	0.01843	0.9531	0.517
	Age	0.3392	0.03605	1.1172	0.208	0.3401	0.02027	1.0488	0.308
Bray-Curtis	Species	2.1719	0.23115	7.3834	**0.001**	2.2328	0.13488	7.098	**0.001**
	Site	1.5346	0.16332	1.7389	**0.001**	1.7422	0.10525	1.8462	**0.001**
	Sex	0.3198	0.03403	1.0871	0.26	0.2932	0.01771	0.9322	0.556
	Age	0.3692	0.03929	1.2552	0.145	0.3319	0.02005	1.0552	0.304
Unweighted unifrac	Species	0.96079	0.34494	12.9127	**0.001**	1.0477	0.24827	15.1989	**0.001**
	Site	0.39258	0.14094	1.7587	**0.025**	0.4091	0.09694	1.9782	**0.006**
	Sex	0.08852	0.03178	1.1896	0.228	0.0627	0.01485	0.9092	0.502
	Age	0.07858	0.02821	1.0561	0.31	0.0811	0.01923	1.1772	0.228
Weighted unifrac	Species	0.56929	0.3133	11.7362	**0.001**	0.46014	0.15965	8.8899	**0.001**
	Site	0.26719	0.14704	1.8361	**0.034**	0.31747	0.11015	2.0445	**0.018**
	Sex	0.04746	0.02612	0.9784	0.401	0.0359	0.01246	0.6936	0.669
	Age	0.10852	0.05972	2.2372	**0.044**	0.10174	0.0353	1.9657	0.071

Dissimilarity Index	Variable	Cecum: Sum of squares	Cecum: R^2	Cecum: F	Cecum: p	Small Intestine: Sum of squares	Small Intestine: R^2	Small Intestine: F	Small Intestine: p
Jaccard	Species	2.1867	0.24652	7.5102	**0.001**	1.8038	0.0873	4.8339	**0.001**
	Site	1.4027	0.15813	1.6058	**0.015**	1.9785	0.09576	1.7674	**0.001**
	Sex	0.2921	0.03293	1.0033	0.346	0.3675	0.01779	0.9848	0.452
	Age	0.3303	0.03724	1.1345	0.237	0.4659	0.02255	1.2486	0.076
Bray-Curtis	Species	2.3726	0.26921	8.4506	**0.001**	2.1944	0.10802	6.2356	**0.001**
	Site	1.3737	0.15586	1.6309	**0.024**	1.928	0.0949	1.8262	**0.001**
	Sex	0.258	0.02928	0.9191	0.476	0.3143	0.01547	0.8931	0.603
	Age	0.3168	0.03595	1.1285	0.259	0.7461	0.03672	2.12	**0.002**
Unweighted unifrac	Species	0.96941	0.37484	14.7491	**0.001**	0.8717	0.12151	6.905	**0.001**
	Site	0.42496	0.16432	2.1552	**0.009**	0.6244	0.08704	1.6487	**0.011**
	Sex	0.05945	0.02299	0.9046	0.425	0.1134	0.01581	0.8987	0.522
	Age	0.08078	0.03123	1.229	0.248	0.1359	0.01894	1.0762	0.329
Weighted unifrac	Species	0.45296	0.28928	8.8961	**0.001**	1.8437	0.17247	10.5428	**0.001**
	Site	0.1951	0.1246	1.2772	0.196	0.6754	0.06318	1.2873	0.239
	Sex	0.03483	0.02225	0.6841	0.685	0.1019	0.00953	0.5828	0.651
	Age	0.06826	0.04359	1.3405	0.22	0.5494	0.0514	3.1419	**0.024**

Table S21: Pairwise PERMANOVA results comparing gut regions between *A. argenteus* and *M. rufocanus*. Bold text indicates statistical significance.

Number of more abundant genera per gut region (All GIT regions included, LEfSe)

Species	Rectum	Colon	Cecum	Small Intestine	Average
A. speciosus	1	0	0	0	0.25
A. argenteus	17	4	11	4	9.75
M. rufocanus	0	0	1	0	0.25
**Average**	6	1.67	4.67	1.33	

Table S22: Number of more abundant genera found in each gut region based on LEfSe analysis when all regions were included.

Number of more abundant genera per gut region (pairwise LEfSe)

	**CE - SI**		**RC - SI**		**CL - SI**		**CE – CL**		**CE - RC**		**RC - CL**	
**Species**	**CE**	**SI**	**RC**	**SI**	**CL**	**SI**	**CE**	**CL**	**CE**	**RC**	**RC**	**CL**
A. speciosus	47	23	44	19	47	22	4	4	5	5	7	5
A. argenteus	39	10	35	7	33	8	1	0	2	2	1	0
M. rufocanus	48	9	53	10	50	20	6	0	1	3	12	0
**Average**	44.67	14	44	12	43.33	16.67	3.67	1.33	2.67	3.33	6.67	1.67

Table S23: Number of more abundant genera found in each gut region based on pairwise LEfSe analysis we SI is small intestine, CE is cecum, CL is colon, and RC is the rectum.

Species	Small intestine		Cecum		Colon		Rectum	
	**Within species all gut regions included**							
	Genus	LDA	Genus	LDA	Genus	LDA	Genus	LDA
A. speciosus	—	—	—	—	—	—	Ruminococcaceae NK4A214	4.32
A. argenteus	Lactobacillus	4.76	Campylobacter	4.60	Ruminiclostridium 9	3.91	Alistipes	4.54
	Streptococcus	4.33	Treponema 2	4.59	Ruminiclostridium	3.84	Lachospiraceae NK4A136	4.34
	Rodentibacter	4.25	Lachnospiraceae UCG_010	3.86	Intestinimonas	3.24	Bacteroides	4.43
	Veillonella	3.48	Oscillibacter	3.94	Oxalobacter	3.45	Rikenellaceae RC9	4.33
			Odoribacter	3.64			*Prevotellaceae UCG_003*	4.11
			Desulfovibrio	3.38			Rikenella	3.95
			Roseburia	3.18			Prevotellaceae Ga6A1	3.76
			Ruminococcaceae UCG_004	3.36			Ruminiclostridium 6	3.59
			Tyzzerella	3.09			Butyricicoccus	3.12
			Ruminiclostridium 5	3.11			Ruminococcaceae UCG_010	3.19
			Acetatifactor	3.01			Ruminococcaceae UCG_014	3.17
							Muribaculum	3.17
							Eubacterium coprostanoligenes	3.10
							Prevotellaceae UCG_001	3.11
							Ruminococcaceae NK4A214	3.15
							Lachnospiraceae UCG_006	3.12
							Lachnospiraceae GCA_900066575	2.96
M. rufocanus	—	—	Treponema 2	4.52	—	—	—	—

Table S24: LEfSe results showing more abundant genera within each gut region when all gut regions were included for *A. speciosus*, *A. argenteus* and *M. rufocanus*. LDA scores shown.

Within species (A. speciosus) pairwise between the upper and lower GIT							
Small Intestine - Colon				Small Intestine - Cecum			
Small Intestine		Colon		Small Intestine		Cecum	
Genus	LDA	Genus	LDA	Genus	LDA	Genus	LDA
Tyzzerella 3	5.31	Helicobacter	4.63	Tyzzerella 3	5.30	Helicobacter	4.62
Escherichia-Shigella	4.58	Alistipes	4.42	Escherichia-Shigella	4.58	Alistipes	4.33
Ureaplasma	4.37	Lachnospiraceae NK4A136	4.28	Ureaplasma	4.44	Oscillibacter	4.27
Lactobacillus	4.00	Rickenellaceae RC9	4.17	Lactobacillus	4.00	Bacteroides	4.26
Streptococcus	3.74	Bacteroides	4.11	Streptococcus	3.92	Lachnospiraceae NK4A136	4.18
Turicibacter	3.60	Oscillibacter	3.96	Delftia	3.63	Prevotellaceae UCG_003	4.13
Rodentibacter	3.30	Prevotellaceae Ga6A1	3.81	Enterococcus	3.56	Ruminiclostridium 9	4.07
Chthoniobacterales	3.26	Ruminiclostridium 9	3.75	Turicibacter	3.55	Treponema 2	3.99
Veillonella	3.21	Treponema 2	3.73	Gemella	3.43	Mucispirillum	3.95
Enterococcus	3.09	Ruminiclostridium	3.68	Rodentibacter	3.37	Rikenellaceae RC9 gut	3.87
Delftia	3.07	Odoribacter	3.59	Veillonella	3.24	Ruminiclostridium	3.84
Acinetobacter	2.69	Ruminiclostridium 6	3.52	Stenotrophomonas	3.15	Prevotellaceae Ga6A1	3.83
Pseudomonas	2.61	Eubacterium, coprostanoligenes	3.50	Bergeyella	3.14	Odoribacter	3.51
Exiguobacterium	2.61	Intestinimonas	3.27	Providencia	2.99	Lachnospiraceae FCS020	3.47
Fusobacterium	2.61	Eubacterium, oxidoreducens	3.26	Carnobacterium	2.87	Intestinimonas	3.46
Bergeyella	2.59	Roseburia	3.23	Acinetobacter	2.86	Ruminiclostridium 6	3.43
Leptotrichia	2.54	Prevotellaceae UCG_001	3.10	Leptotrichia	2.78	Muribaculum	3.24
Providencia	2.53	Ruminococcaceae UCG_014	3.10	Serratia	2.78	Prevotellaceae UCG_001	3.20
Staphylococcus	2.46	Acetatifactor	3.09	Staphylococcus	2.75	Butyrivibrio	3.18
Carnobacterium	2.44	Muribaculum	2.98	Enterorhabdus	2.73	Eubacterium coprostanoligenes	3.18
Stenotrophomonas	2.37	Lachnospiraceae, GCA_900066575	2.97	Pseudomonas	2.73	Candidatus Soleaferrea	3.17
Serratia	2.34	Tyzzerella	2.97	Fusobacterium	2.64	Ruminococcaceae UCG_014	3.12
		Lachnospiraceae FCS020	2.93	Corynebacterium 1	2.60	Rikenella	3.09
		Butyrivibrio	2.88			Roseburia	3.08
		Rikenella	2.86			Lachnospiraceae UCG_010	3.03
		Gastranaerophilales	2.83			Oxalobacter	3.02
		Ruminococcaceae UCG_010	2.77			Eubacterium nodatum	3.00
		Blautia	2.70			Gastranaerophilales	2.98
		Eubacterium brachy	2.67			Tyzzerella	2.98
		Oxalobacter	2.66			Acetatifactor	2.93
		Lachnospiraceae NC2004	2.64			Lachnospiraceae, GCA_900066575	2.87
		Lachnospiraceae UCG_006	2.54			Candidatus Saccharimonas	2.84
		Lachnoclostridium	2.52			Ruminococcaceae UCG_010	2.83
		Parabacteroides	2.51			Ruminococcaceae NK4A214	2.81
		Desulfovibrio	2.48			Ruminococcaceae UCG_004	2.80
		Ruminococcus 1	2.45			Bilophila	2.71
		Butyricicoccus	2.44			Parabacteroides	2.71
		Lachnospiraceae UCG_010	2.43			Ruminococcaceae UCG_009	2.71
		Ruminococcaceae NK4A214	2.42			Desulfovibrio	2.68
		Candidatus Saccharimonas	2.39			Lachnoclostridium	2.67
		Eubacterium nodatum	2.38			Lachnospiraceae UCG_006	2.66
		Ruminococcaceae UCG_004	2.36			Lachnospiraceae NC2004	2.66
		Bilophila	2.35			Ruminococcus 1	2.66
		Ruminococcaceae UCG_002	2.35			Peptococcus	2.66
		Ruminococcaceae UCG_009	2.28			Ruminococcaceae UCG_002	2.62
		Cerasicoccus	2.20			Blautia	2.58
		Peptococcus	2.15			Butyricicoccus	2.52

Table S25: More abundant bacterial genera as compared between the upper and lower GIT in *A. speciosus* based on pairwise LEfSe analysis. LDA scores shown.

Within species (A. speciosus) pairwise between the upper and lower GIT			
**Small Intestine - Rectum**			
**Small Intestine**		**Rectum**	
**Genus**	**LDA**	**Genus**	**LDA**
Tyzzerella 3	5.30	Alistipes	4.44
Ureaplasma	4.39	Bacteroides	4.38
Lactobacillus	3.94	Lachnospiraceae NK4A136	4.36
Turicibacter	3.55	Helicobacter	4.27
Rodentibacter	3.31	Prevotellaceae UCG_003	4.15
Veillonella	3.24	Rikenellaceae RC9	4.13
Delftia	3.23	Prevotellaceae Ga6A1	3.97
Enterococcus	3.06	Oscillibacter	3.88
Providencia	2.91	Treponema 2	3.87
Carnobacterium	2.71	Mucispirillum	3.77
Acinetobacter	2.62	Ruminiclostridium 9	3.77
Serratia	2.61	Ruminiclostridium	3.68
Leptotrichia	2.60	Ruminiclostridium 6	3.68
Fusobacterium	2.59	Odoribacter	3.53
Pseudomonas	2.56	Rikenella	3.49
Bergeyella	2.54	Butyrivibrio	3.47
Corynebacterium 1	2.50	Prevotellaceae UCG_001	3.38
Staphylococcus	2.45	Muribaculum	3.31
Stenotrophomonas	2.40	Intestinimonas	3.30
		Eubacterium coprostanoligenes	3.25
		Candidatus Saccharimonas	3.15
		Roseburia	3.02
		Lachnospiraceae UCG_010	2.81
		Lachnospiraceae, GCA_900066575	2.79
		Candidatus Soleaferrea	2.78
		Tyzzerella	2.77
		Parabacteroides	2.73
		Peptococcus	2.71
		Acetatifactor	2.69
		Ruminococcaceae NK4A214	2.69
		Eubacterium nodatum	2.69
		Ruminococcus 1	2.66
		Lachnoclostridium	2.64
		Lachnospiraceae NC2004	2.58
		Desulfovibrio	2.56
		Ruminococcaceae UCG_002	2.55
		Ruminococcaceae UCG_009	2.53
		Lachnospiraceae UCG_006	2.53
		Ruminococcaceae UCG_004	2.51
		Bilophila	2.46
		Butyricicoccus	2.46
		Blautia	2.44
		Oxalobacter	2.39

Table S25 (continued): More abundant bacterial genera as compared between the upper and lower GIT in *A. speciosus* based on pairwise LEfSe analysis. LDA scores shown.

Within species (*A. speciosus*) pairwise of the lower GIT											
**Cecum - Rectum**				**Colon - Cecum**				**Colon - Rectum**			
**Cecum**		**Rectum**		**Colon**		**Cecum**		**Colon**		**Rectum**	
**Genus**	**LDA**	**Genus**	**LDA**	**Genus**	**LDA**	**Genus**	**LDA**	**Genus**	**LDA**	**Genus**	**LDA**
Helicobacter	4.35	Streptococcus	3.93	Lactobacillus	3.76	Oscillibacter	3.97	Helicobacter	4.32	Rikenella	3.35
Oscillibacter	4.09	Gemella	3.18	Streptococcus	3.53	Ruminiclostridium 9	3.78	Tyzzerella 3	3.87	Prevotellaceae UCG_001	3.07
Ruminiclostridium 9	3.80	Ruminococcaceae NK4A214	2.29	Gemella	2.58	Prevotellaceae UCG_001	3.00	Acetatifactor	2.87	Muribaculum	3.05
Ruminiclostridium	3.42	Ruminococcus	2.28	Corynebacterium 1	2.08	Ruminococcaceae NK4A214	2.05	Lachnospiraceae UCG_010	2.60	Candidatus Saccharimonas	3.02
Lachnoclostridium	2.18	Enterorhabdus	2.18					Cerasicoccus	2.32	Parasutterella	2.99
										Ruminococcaceae NK4A214	2.67
										Ruminococcaceae UCG_002	2.53

Table S26: More abundant bacterial genera as compared between the different regions of the lower GIT in *A. speciosus* based on pairwise LEfSe analysis. LDA scores shown.

Within species (A. argenteus) pairwise between the upper and lower GIT							
**Small Intestine - Colon**				**Small Intestine - Cecum**			
**Small Intestine**		**Colon**		**Small Intestine**		**Cecum**	
**Genus**	**LDA**	**Genus**	**LDA**	**Genus**	**LDA**	**Genus**	**LDA**
Roseburia	3.06	Lactobacillus	4.69	Mycoplasma	5.05	Treponema 2	4.66
Tyzzerella	3.07	Streptococcus	4.33	Lactobacillus	4.83	Campylobacter	4.61
Lachnospiraceae, GCA_900066575	3.12	Rodentibacter	4.28	Streptococcus	4.34	Alistipes	4.47
Ruminiclostridium 6	3.13	Pseudomonas	3.91	Rodentibacter	4.27	Bacteroides	4.25
Intestinimonas	3.26	Coxiella	3.90	Veillonella	3.48	Rikenellaceae RC9	4.20
Eubacterium coprostanoligenes	3.26	Gemella	3.64	Leptotrichia	3.27	Prevotellaceae UCG_003	4.13
Parasutterella	3.32	Veillonella	3.54	Bifidobacterium	3.16	Lachnospiraceae NK4A136	4.06
Prevotellaceae UCG_001	3.35	Leptotrichia	3.36	Coxiella	3.13	Oscillibacter	3.92
Muribaculum	3.38			Pseudomonas	3.09	Ruminiclostridium 9	3.75
Ruminococcaceae NK4A214	3.45			Gemella	2.90	Prevotellaceae Ga6A1	3.71
Ruminococcaceae UCG_010	3.47					Odoribacter	3.67
Ruminiclostridium 5	3.48					Rikenella	3.66
Oxalobacter	3.50					Ruminiclostridium 6	3.51
Odoribacter	3.52					Ruminiclostridium	3.48
Prevotellaceae Ga6A1	3.56					Eubacterium nodatum	3.34
Rikenella	3.67					Lachnospiraceae UCG_010	3.33
Ruminococcaceae UCG_004	3.70					Ruminococcaceae UCG_004	3.27
Acetatifactor	3.76					Oxalobacter	3.24
Ruminococcaceae UCG_014	3.76					Roseburia	3.21
Ruminiclostridium	3.80					Blautia	3.21
Ruminococcaceae UCG_009	3.84					Butyricicoccus	3.19
Ruminiclostridium 9	3.87					Ruminococcaceae UCG_010	3.14
Oscillibacter	3.88					Acetatifactor	3.11
Prevotellaceae UCG_003	3.95					Desulfovibrio	3.09
Bacteroides	3.98					Lachnoclostridium	3.04
Treponema 2	4.01					Eubacterium coprostanoligenes	3.00
Butyricicoccus	4.03					Tyzzerella	3.00
Eubacterium nodatum	4.05					Intestinimonas	3.00
Rikenellaceae RC9	4.07					Muribaculum	2.99
Lachnospiraceae NK4A136	4.20					Ruminiclostridium 5	2.98
Campylobacter	4.38					Prevotellaceae UCG_001	2.95
Alistipes	4.51					Ruminococcaceae NK4A214	2.94
						Parasutterella	2.92
						Ruminococcaceae UCG_014	2.92
						Candidatus Saccharimonas	2.91
						Bilophila	2.90
						Lachnospiraceae, GCA_900066575	2.83
						Lachnospiraceae UCG_006	2.83
						Ruminococcus 1	2.81

Table S27: More abundant bacterial genera as compared between the upper and lower GIT in *A. argenteus* based on pairwise LEfSe analysis. LDA scores shown.

Within species (A. argenteus) pairwise between the upper and lower GIT			
**Small Intestine - Rectum**			
**Small Intestine**		**Rectum**	
**Genus**	**LDA**	**Genus**	**LDA**
Lactobacillus	4.76	Alistipes	4.54
Streptococcus	4.27	Bacteroides	4.35
Veillonella	3.52	Lachnospiraceae NK4A136	4.30
Coxiella	3.36	Rikenellaceae RC9	4.30
Pseudomonas	3.34	Campylobacter	4.29
Leptotrichia	3.32	Treponema 2	4.22
Gemella	2.99	Prevotellaceae UCG_003	4.11
		Rikenella	3.87
		Prevotellaceae Ga6A1	3.79
		Oscillibacter	3.72
		Lachnospiraceae UCG_010	3.68
		Ruminiclostridium 6	3.57
		Ruminiclostridium 9	3.54
		Eubacterium nodatum	3.53
		Ruminiclostridium	3.51
		Coriobacteriaceae UCG_002	3.51
		Odoribacter	3.46
		Desulfovibrio	3.20
		Muribaculum	3.20
		Ruminococcaceae UCG_004	3.17
		Eubacterium coprostanoligenes	3.15
		Acetatifactor	3.15
		Ruminococcaceae UCG_014	3.14
		Oxalobacter	3.13
		Prevotellaceae UCG_001	3.10
		Roseburia	3.05
		Intestinimonas	3.05
		Candidatus Saccharimonas	3.05
		Ruminococcaceae NK4A214	3.04
		Ruminiclostridium 5	2.97
		Lachnospiraceae, GCA_900066575	2.92
		Butyricicoccus	2.92
		Ruminococcaceae UCG_010	2.86
		Tyzzerella	2.80
		Lachnospiraceae UCG_006	2.72

Table S27 (continued): More abundant bacterial genera as compared between the upper and lower GIT in *A. argenteus* based on pairwise LEfSe analysis. LDA scores shown.

Within species (*A. argenteus*) pairwise of the lower GIT											
**Cecum - Rectum**				**Colon - Cecum**				**Colon - Rectum**			
**Cecum**		**Rectum**		**Colon**		**Cecum**		**Colon**		**Rectum**	
**Genus**	**LDA**	**Genus**	**LDA**	**Genus**	**LDA**	**Genus**	**LDA**	**Genus**	**LDA**	**Genus**	**LDA**
Campylobacter	4.31	Rodentibacter	3.89	—	—	Desulfovibrio	2.54	—	—	Rodentibacter	3.89

Table S28: More abundant bacterial genera as compared between the different regions of the lower GIT in *A. argenteus* based on pairwise LEfSe analysis. LDA scores shown.

Within species (M. rufocanus) pairwise between the upper and lower GIT							
**Small Intestine - Colon**				**Small Intestine - Cecum**			
**Small Intestine Genus**	**LDA**	**Colon Genus**	**LDA**	**Small Intestine Genus**	**LDA**	**Cecum Genus**	**LDA**
Ureaplasma	4.69	Lachnospiraceae NK4A136	4.33	Ureaplasma	4.67	Treponema 2	4.49
Lactobacillus	4.46	Eubacterium oxidoreducens	4.32	Lactobacillus	4.43	Lachnospiraceae NK4A136	4.13
Helicobacter	4.03	Alistipes	4.15	Helicobacter	3.94	Ruminiclostridium	4.08
Rickettsia	3.99	Treponema 2	4.14	Desulfovibrio	3.87	Alistipes	4.05
Rodentibacter	3.84	Ruminiclostridium	4.00	Escherichia-Shigella	3.34	Oscillibacter	3.93
Desulfovibrio	3.83	Anaerovorax	3.79	Veillonella	3.28	Ruminiclostridium 9	3.71
Enterococcus	3.81	Ruminiclostridium 9	3.72	Streptococcus	2.76	Odoribacter	3.69
Exiguobacterium	3.72	Odoribacter	3.71	Coxiella	2.37	Ruminiclostridium 6	3.65
Staphylococcus	3.53	Oscillibacter	3.70	Carnobacterium	2.37	Eubacterium coprostanoligenes	3.53
Coxiella	3.36	Lachnospiraceae UCG_001	3.66			Ruminococcaceae, UBA1819	3.33
Escherichia-Shigella	3.35	Lachnospiraceae UCG_010	3.60			Candidatus Saccharimonas	3.26
Ehrlichia	3.32	Harryflintia	3.59			Eubacterium xylanophilum	3.15
Veillonella	3.32	Ruminococcaceae UCG_013	3.55			Millionella	3.14
Candidatus Saccharimonas	3.26	Ruminiclostridium 6	3.53			Rikenella	3.10
Carnobacterium	3.17	Ruminococcaceae V9D2013	3.52			Roseburia	3.10
Streptococcus	3.16	Cerasicoccus	3.44			Ruminococcaceae UCG_010	3.07
Enterorhabdus	3.14	Ruminococcaceae, UBA1819	3.39			Ruminococcaceae UCG_014	3.07
Acinetobacter	3.00	Erysipelotrichaceae UCG_004	3.36			Ruminococcaceae NK4A214	3.05
Fusobacterium	2.89	Eubacterium coprostanoligenes	3.36			Eubacterium brachy	3.03
		Ruminococcaceae GCA_900066225	3.31			Peptococcus	3.03
		Bilophila	3.28			Intestinimonas	3.03
		Roseburia	3.27			Mucispirillum	2.96
		Lachnospiraceae, GCA_900066575	3.26			Ruminococcaceae UCG_009	2.94
		Lachnospiraceae NK4B4	3.26			Ruminococcus 1	2.94
		Ruminiclostridium 5	3.25			Acetatifactor	2.91
		Ruminococcaceae UCG_014	3.24			Tyzzerella	2.91
		Candidatus Soleaferrea	3.23			Lachnospiraceae UCG_010	2.84
		Mucispirillum	3.22			Ruminiclostridium 5	2.83
		Intestinimonas	3.22			Clostridiales, Family XIII, AD3011	2.79
		Ruminococcaceae UCG_010	3.21			Brachyspira	2.76
		Oxalobacter	3.19			Lachnospiraceae, ASF356	2.75
		Rikenella	3.17			Ruminococcaceae UCG_005	2.68
		Ruminococcaceae NK4A214	3.17			Lachnospiraceae NC2004	2.65
		Fournierella	3.17			Fournierella	2.62
		Butyricicoccus	3.17			Bilophila	2.57
		Eubacterium xylanophilum	3.14			Pygmaiobacter	2.56
		Millionella	3.10			Eubacterium ruminantium	2.56
		Acetatifactor	3.08			Ruminococcaceae UCG_004	2.53
		Ruminococcaceae UCG_004	3.07			Eubacterium oxidoreducens	2.52
		Ruminococcus 1	3.03			Harryflintia	2.51
		Pygmaiobacter	3.02			Candidatus Soleaferrea	2.48
		Ruminococcaceae UCG_009	3.00			Oxalobacter	2.48
		Ruminococcaceae UCG_005	2.99			Butyrivibrio	2.48
		Spirochaetaceae, GWE2_31_10	2.99			Lachnospiraceae, GCA_900066575	2.37
		Peptococcus	2.98			Lachnoclostridium	2.36
		Butyrivibrio	2.93			Ruminococcaceae UCG_002	2.35
		Ruminococcaceae UCG_002	2.92			Ruminococcaceae V9D2013	2.33
		Brachyspira	2.89			Angelakisella	2.30
		Tyzzerella	2.87			Erysipelotrichaceae UCG_004	2.19

Table S29: More abundant bacterial genera as compared between the upper and lower GIT in *M. rufocanus* based on pairwise LEfSe analysis. LDA scores shown.

Within species (M. rufocanus) pairwise between the upper and lower GIT			
Small Intestine - Rectum			
Small Intestine		Rectum	
**Genus**	**LDA**	**Genus**	**LDA**
Ureaplasma	4.68	Lachnospiraceae UCG_010	4.48
Lactobacillus	4.43	Lachnospiraceae NK4A136	4.35
Helicobacter	4.05	Treponema 2	4.34
Rodentibacter	3.79	Alistipes	3.98
Escherichia-Shigella	3.36	Ruminiclostridium	3.98
Veillonella	3.29	Ruminococcus 1	3.86
Carnobacterium	3.09	Anaerofilum	3.84
Streptococcus	2.93	Ruminiclostridium 6	3.80
Coxiella	2.80	Eubacterium xylanophilum	3.70
Fusobacterium	2.69	Clostridiales, Family XIII, UCG_001	3.67
		Ruminiclostridium 9	3.63
		Ruminococcaceae, UBA1819	3.62
		Oscillibacter	3.55
		Odoribacter	3.55
		Ruminococcaceae UCG_004	3.54
		Lachnospiraceae FCS020	3.51
		Lachnospiraceae, ASF356	3.49
		Erysipelotrichaceae UCG_004	3.49
		Ruminiclostridium 1	3.48
		Candidatus Saccharimonas	3.43
		Ruminococcaceae UCG_014	3.39
		Eubacterium coprostanoligenes	3.35
		Ruminococcaceae UCG_010	3.34
		Clostridiales, Family XIII, AD3011	3.32
		Acetatifactor	3.31
		Eubacterium oxidoreducens	3.24
		Ruminiclostridium 5	3.22
		Intestinimonas	3.19
		Lachnospiraceae NC2004	3.19
		Ruminococcaceae NK4A214	3.18
		Candidatus Soleaferrea	3.13
		Bilophila	3.12
		Rikenella	3.12
		Peptococcus	3.11
		Ruminococcaceae GCA_900066225	3.09
		Pygmaiobacter	3.08
		Lachnospiraceae NK4B4	3.07
		Millionella	3.05
		Ruminococcaceae UCG_009	2.99
		Ruminococcaceae UCG_005	2.99
		Brachyspira	2.93
		Fournierella	2.93
		Ruminococcaceae UCG_013	2.92
		Mucispirillum	2.92
		Butyrivibrio	2.92
		Lachnoclostridium	2.91
		Eubacterium ruminantium	2.90
		Lachnospiraceae, GCA_900066575	2.90
		Ruminococcaceae UCG_002	2.89
		Tyzzerella	2.86
		Oxalobacter	2.86
		Harryflintia	2.66
		Quinella	2.62

Table S29 (continued): More abundant bacterial genera as compared between the upper and lower GIT in *M. rufocanus* based on pairwise LEfSe analysis. LDA scores shown.

Within species (*M. rufocanus*) pairwise of the lower GIT

Cecum - Rectum				Colon - Cecum						Colon - Rectum			
**Cecum**		**Rectum**		**Colon**		**Cecum**		**Colon**		**Rectum**			
**Genus**	**LDA**	**Genus**	**LDA**	**Genus**	**LDA**	**Genus**	**LDA**	**Genus**	**LDA**	**Genus**	**LDA**		
Oscillibacter	3.64	Ruminococcus 1	3.87	Treponema 2	4.29	—	—	—	—	Treponema 2	4.00		
		Eubacterium xylanophilum	3.60	Oscillibacter	3.46					Ruminococcus 1	3.86		
		Pygmaiobacter	2.74	Clostridiales, FamilyXIII, AD3011	3.01					Eubacterium xylanophilum	3.55		
				Peptococcus	2.88					Ruminiclostridium 6	3.47		
				Brachyspira	2.76					Coxiella	3.39		
				Ruminococcaceae, UCG_005	2.55					Bacteroides	3.30		
										Acetatifactor	3.05		
										Ruminiclostridium 5	2.89		
										Pygmaiobacter	2.71		
										Peptococcus	2.57		
										Ruminococcaceae, UCG_005	2.56		
										Clostridiales, Family XIII, AD3011	2.12		

Table S30: More abundant bacterial genera as compared between the different regions of the lower GIT in *M. rufocanus* based on pairwise LEfSe analysis. LDA scores shown.

Number of more abundant genera per host species (all species included, LEfSe)

	A. speciosus	*A. argenteus*	*M. rufocanus*
**Small Intestine**	17	8	10
**Cecum**	14	9	15
**Colon**	20	8	17
**Rectum**	15	6	16
**Average**	16.5	7.75	14.5

Table S31: Number of more abundant genera when gut region is compared among species based on LEfSe analysis when all species were included.

Number of more abundant genera per host species (pairwise LEfSe)

	A. speciosus* - *A. argenteus		***A. speciosus* - *M. rufocanus***		***A. argenteus* - *M. rufocanus***	
	A. speciosus	***A. argenteus***	***A. speciosus***	***M. rufocanus***	***A. argenteus***	***M. rufocanus***
**Small Intestine**	6	**28**	26	21	**41**	7
**Cecum**	9	7	24	30	22	**28**
**Colon**	**12**	1	**32**	31	18	23
**Rectum**	8	3	22	**32**	17	22
**Average**	8.75	9.75	26	28.5	24.5	20

Table S32: Number of more abundant genera when gut region is compared among species based on pairwise LEfSe analysis.

Gut Region	Among species, all three host species included					
	A. speciosus		*A. argenteus*		*M. rufocanus*	
	**Genus**	**LDA**	**Genus**	**LDA**	**Genus**	**LDA**
**Small intestine**	Tyzzerella 3	5.34	Escherichia-Shigella	4.95	Ureaplasma	4.64
	Bergeyella	3.90	Lactobacillus	4.70	Ruminococcaceae, UCG_005	4.00
	Ruminiclostridium 5	3.75	Streptococcus	4.42	Desulfovibrio	3.93
	Corynebacterium 1	3.67	Gemella	3.70	Ruminococcaceae, UCG_010	3.92
	Turicibacter	3.59	Providencia	3.36	Candidatus Saccharimonas	3.60
	Serratia	3.55	Leptotrichia	3.29	Lachnospiraceae NK4A136	3.47
	Bacteroides	3.30	Treponema 2	3.20	Ruminiclostridium	3.27
	Mucispirillum	3.27	Coxiella	2.82	Ruminiclostridium 6	3.25
	Prevotellaceae, UCG_001	3.20			Roseburia	3.23
	Muribaculum	3.16			Ruminococcus 1	3.21
	Rikenellaceae RC9	3.15				
	Prevotellaceae Ga6A1	3.14				
	Parasutterella	3.10				
	Odoribacter	3.05				
	Prevotellaceae, UCG_003	3.00				
	Rikenella	2.98				
	Fusobacterium	2.74				
**Cecum**	Helicobacter	4.58	Treponema 2	4.54	Ruminiclostridium	3.96
	Bacteroides	4.32	Alistipes	4.25	Candidatus Saccharimonas	3.61
	Oscillibacter	4.03	Rikenellaceae RC9	4.20	Ruminiclostridium 6	3.38
	Prevotellaceae, UCG_003	4.01	Escherichia-Shigella	3.92	Clostridiales, Family XIII, AD3011	3.33
	Prevotellaceae Ga6A1	3.94	Brachyspira	3.61	Ruminococcaceae, UCG_005	3.25
	Mucispirillum	3.94	Streptococcus	3.47	Eubacterium xylanophilum	3.22
	Tyzzerella 3	3.85	Turicibacter	3.12	Ruminococcaceae, UCG_013	3.22
	Ruminiclostridium 9	3.79	Parasutterella	2.96	Peptococcus	3.18
	Muribaculum	3.31	Lachnospiraceae, UCG_006	2.88	Ruminococcaceae, UCG_009	3.13
	Prevotellaceae, UCG_001	3.29			Ruminococcaceae, UCG_010	3.08
	Eubacterium nodatum	3.17			Ruminococcaceae NK4A214	3.07
	Lachnospiraceae, GCA_900066575	2.86			Ruminococcus 1	3.02
	Butyricicoccus	2.81			Candidatus Soleaferrea	3.00
	Bilophila	2.81			Desulfovibrio	2.98
					Oxalobacter	2.86

Table S33: More abundant bacterial genera within the small intestine and cecum of each host species when all three hosts were included in LEfSe analysis. LDA scores shown.

Gut Region	**Among species, all three host species included**					
	A. speciosus		***A. argenteus***		***M. rufocanus***	
	**Genus**	**LDA**	**Genus**	**LDA**	**Genus**	**LDA**
**Colon**	Helicobacter	4.62	Mycoplasma	4.56	Treponema 2	3.92
	Rikenellaceae RC9	4.22	Alistipes	4.26	Clostridiales, Family XIII AD3011	3.88
	Bacteroides	4.19	Escherichia-Shigella	4.08	Ruminiclostridium	3.76
	Tyzzerella 3	4.04	Prevotella 9	3.85	Candidatus Saccharimonas	3.52
	Mucispirillum	3.89	Prevotellaceae, UCG_003	3.85	Ruminiclostridium 6	3.47
	Prevotellaceae Ga6A1	3.86	Rikenella	3.59	Oxalobacter	3.35
	Streptococcus	3.69	Parasutterella	3.08	Ruminococcaceae, UCG_005	3.28
	Eubacterium nodatum	3.44	Brachyspira	2.90	Desulfovibrio	3.27
	Enterorhabdus	3.22			Eubacterium xylanophilum	3.24
	Ruminiclostridium 5	3.20			Ruminococcaceae, UCG_010	3.21
	Muribaculum	3.18			Ruminococcaceae NK4A214	3.12
	Butyricicoccus	3.15			Ruminococcaceae, UCG_009	3.07
	Prevotellaceae, UCG_001	3.13			Ruminococcaceae, UCG_013	3.06
	Lachnospiraceae, UCG_006	3.07			Anaeroplasma	3.05
	Blautia	2.99			Peptococcus	3.03
	Lachnoclostridium	2.92			Ruminococcus 1	2.99
	Lachnospiraceae, GCA_900066575	2.92			Candidatus Soleaferrea	2.81
	Cerasicoccus	2.91				
	Tyzzerella	2.91				
	Turicibacter	2.83				
**Rectum**	Bacteroides	4.41	Mycoplasma	4.41	Treponema 2	4.15
	Helicobacter	4.26	Alistipes	4.39	Ruminococcus 1	3.85
	Escherichia-Shigella	4.11	Rikenellaceae, RC9 gut	4.32	Ruminiclostridium	3.84
	Prevotellaceae, UCG_003	4.06	Rikenella	3.93	Candidatus Saccharimonas	3.76
	Prevotellaceae Ga6A1	4.05	Lachnospiraceae, UCG_006	3.19	Eubacterium xylanophilum	3.73
	Streptococcus	3.96	Brachyspira	3.02	Peptococcus	3.73
	Mucispirillum	3.73			Candidatus Soleaferrea	3.42
	Tyzzerella 3	3.58			Clostridiales, Family XIII, AD3011	3.42
	Eubacterium nodatum	3.53			Ruminiclostridium 6	3.41
	Prevotellaceae, UCG_001	3.47			Acetatifactor	3.31
	Muribaculum	3.39			Ruminococcaceae, UCG_010	3.29
	Butyricicoccus	3.03			Desulfovibrio	3.29
	Parasutterella	3.00			Ruminococcaceae, UCG_009	3.20
	Turicibacter	2.90			Ruminococcaceae NK4A214	3.19
	Parabacteroides	2.82			Oxalobacter	3.09
					Anaeroplasma	3.06

Table S33 (continued): More abundant bacterial genera within the colon and rectum of each host species when all three hosts were included in LEfSe analysis. LDA scores shown.

Small Intestine							
A. speciosus - A. argenteus				***A. speciosus - M. rufocanus***			
A. speciosus		***A. argenteus***		***A. speciosus***		***M. rufocanus***	
**Genus**	**LDA**	**Genus**	**LDA**	**Genus**	**LDA**	**Genus**	**LDA**
Tyzzerella 3	5.32	Mycoplasma	4.81	Tyzzerella 3	5.33	Ureaplasma	4.40
Ureaplasma	4.33	Lactobacillus	4.74	Escherichia-Shigella	4.65	Lactobacillus	4.31
Ruminiclostridium 5	3.73	Streptococcus	4.25	Streptococcus	3.99	Desulfovibrio	3.95
Prevotellaceae Ga6A1	3.19	Rodentibacter	4.15	Porphyromonas	3.97	Candidatus Saccharimonas	3.65
Fusobacterium	2.57	Lachnospiraceae NC2004	3.50	Ruminiclostridium 5	3.73	Ruminococcaceae, UCG_010	3.55
Mucispirillum	2.30	Minicystis	3.48	Delftia	3.67	Clostridiales, Family XIII, AD3011	3.53
		Chryseobacterium	3.43	Turicibacter	3.59	Rickettsia	3.51
		Pelosinus	3.33	Bacteroidales RF16	3.52	Ruminococcaceae, UCG_005	3.48
		Gemmatimonas	3.33	Anaerobacillus	3.51	Lachnospiraceae NK4A136	3.38
		Sphingomonas	3.26	Faecalibaculum	3.33	Fournierella	3.28
		Haliangium	3.23	Gemella	3.29	Peptococcus	3.25
		Phyllobacterium	3.20	Bacteroides	3.28	Ruminococcaceae, UBA1819	2.94
		Aquicella	3.20	Corynebacterium 1	3.20	Lachnospiraceae NC2004	2.88
		Variovorax	3.18	Serratia	3.12	Ruminococcus 1	2.86
		Sandaracinus	3.13	Rikenellaceae RC9	3.10	Roseburia	2.84
		Phaselicystis	3.11	Prevotellaceae Ga6A1	3.09	Millionella	2.74
		Leptotrichia	3.11	Parasutterella	3.00	Ruminiclostridium 6	2.66
		Methylobacterium	3.00	Bergeyella	2.95	Eubacterium xylanophilum	2.65
		Bifidobacterium	2.88	Rikenella	2.92	Ruminiclostridium	2.61
		Bradyrhizobium	2.86	Acinetobacter	2.83	Coxiella	2.57
		Ilumatobacter	2.83	Treponema 2	2.82	Candidatus Cardinium	2.53
		Streptomyces	2.75	Muribaculum	2.79		
		Paludibacter	2.72	Prevotellaceae, UCG_001	2.77		
		Campylobacter	2.59	Prevotellaceae, UCG_003	2.73		
		Coxiella	2.56	Leptotrichia	2.62		
		Brachyspira	2.50	Odoribacter	2.57		
		Gemella	2.28	Fusobacterium	2.30		

Table S34: More abundant bacterial genera within the small intestine of each host species based on LEfSe analysis between *A. speciosus* and either *A. argenteus* or *M. rufocanus*. LDA scores shown.

Small Intestine			
M. rufocanus - A. argenteus			
M. rufocanus		*A. argenteus*	
**Genus**	**LDA**	**Genus**	**LDA**
Ureaplasma	4.63	Escherichia-Shigella	5.02
Desulfovibrio	3.91	Mycoplasma	4.99
Candidatus Saccharimonas	3.63	Spirochaeta 2	4.82
Enterorhabdus	2.62	Streptococcus	4.42
Ruminiclostridium 6	2.60	Rodentibacter	4.02
Eubacterium xylanophilum	2.52	Turicibacter	3.52
Ruminococcus 1	2.48	Bradyrhizobium	3.36
		Ilumatobacter	3.29
		Prevotellaceae, UCG_001	3.24
		Leptotrichia	3.19
		Variovorax	3.18
		Paludibacter	3.07
		Candidatus Xiphinematobacter	3.04
		Pirellula	3.01
		Gemmatimonas	2.98
		Phaselicystis	2.94
		Gaiella	2.93
		Bifidobacterium	2.81
		Devosia	2.74
		Sandaracinus	2.67
		Prevotella 9	2.55
		Bacteroides	2.54
		Campylobacter	2.47
		Providencia	2.45
		Prevotellaceae, UCG_003	2.44
		Ilumatobacteraceae, CL500_29 marine	2.44
		Weissella	2.43
		Phyllobacterium	2.41
		Pelosinus	2.36
		Gemella	2.32
		Parasutterella	2.31
		Coxiella	2.28
		Muribaculum	2.19
		Haliangium	2.18
		Streptomyces	2.16
		Pseudomonas	2.15
		Corynebacterium 1	2.15
		Rikenellaceae RC9	2.12
		Methylobacterium	2.10
		Brochothrix	2.00

Table S34 (continued): More abundant bacterial genera within the small intestine of each host species based on LEfSe analysis between *M. rufocanus* and *A. argenteus*.

Cecum							
A. speciosus - A. argenteus				***A. speciosus - M. rufocanus***			
A. speciosus		***A. argenteus***		***A. speciosus***		***M. rufocanus***	
**Genus**	**LDA**	**Genus**	**LDA**	**Genus**	**LDA**	**Genus**	**LDA**
Helicobacter	4.46	Campylobacter	4.62	Helicobacter	4.58	Treponema 2	4.39
Oscillibacter	4.02	Treponema 2	4.54	Bacteroides	4.31	Ruminiclostridium	3.76
Mucispirillum	3.93	Brachyspira	3.69	Alistipes	4.07	Candidatus Saccharimonas	3.55
Tyzzerella 3	3.86	Rikenella	3.52	Oscillibacter	4.02	Ruminiclostridium 6	3.33
Ruminiclostridium 9	3.81	Streptococcus	3.33	Rikenellaceae RC9 gut	3.98	Ruminococcaceae, UBA1819	3.32
Ruminiclostridium	3.62	Oxalobacter	2.92	Prevotellaceae, UCG_003	3.97	Eubacterium xylanophilum	3.21
Ureaplasma	3.22	Rickettsia	2.92	Prevotellaceae, Ga6A1	3.96	Millionella	3.17
Ruminococcaceae, UCG_014	3.07			Mucispirillum	3.90	Peptococcus	3.05
Ruminococcaceae, UCG_009	2.92			Ruminiclostridium 9	3.82	Erysipelotrichaceae, UCG_004	2.99
				Tyzzerella 3	3.81	Ruminococcaceae, UCG_010	2.98
				Escherichia-Shigella	3.79	Ruminococcaceae NK4A214	2.97
				Muribaculum	3.33	Eubacterium oxidoreducens	2.94
				Intestinimonas	3.27	Desulfovibrio	2.90
				Prevotellaceae, UCG_001	3.24	Ruminococcaceae, UCG_009	2.89
				Streptococcus	3.19	Ruminococcus 1	2.88
				Eubacterium nodatum	2.98	Ruminococcaceae, V9D2013	2.83
				Lachnospiraceae, GCA_900066575	2.92	Ruminococcaceae, UCG_013	2.81
				Lachnospiraceae, UCG_006	2.84	Ruminococcaceae, UCG_005	2.79
				Parasutterella	2.81	Brachyspira	2.79
				Lachnoclostridium	2.75	Anaerostipes	2.79
				Parabacteroides	2.74	Lachnospiraceae NK4B4	2.78
				Bilophila	2.74	Clostridiales, Family XIII, AD3011	2.75
				Butyricicoccus	2.72	Pygmaiobacter	2.73
				Coriobacteriaceae, UCG_002	2.58	Eubacterium ruminantium	2.72
						Fournierella	2.69
						Candidatus Soleaferrea	2.61
						Quinella	2.61
						Oxalobacter	2.59
						Harryflintia	2.56
						Ruminococcaceae, GCA_900066225	2.39

Table S35: More abundant bacterial genera within the cecum of each host species based on LEfSe analysis between *A. speciosus* and either *A. argenteus* or *M. rufocanus*. LDA scores shown.

Cecum			
M. rufocanus - A. argenteus			
M. rufocanus		***A. argenteus***	
**Genus**	**LDA**	**Genus**	**LDA**
Ruminiclostridium	3.97	Bacteroides	4.30
Candidatus Saccharimonas	3.60	Alistipes	4.25
Ruminococcaceae V9D2013	3.46	Rikenellaceae RC9 gut	4.21
Clostridiales vadinBB60	3.39	Prevotellaceae, UCG_003	4.00
Tyzzerella 3	3.39	Prevotella 9	3.94
Ruminiclostridium 6	3.36	Mycoplasma	3.94
Erysipelotrichaceae, UCG_004	3.34	Escherichia-Shigella	3.92
Ruminococcaceae, UBA1819	3.31	Prevotellaceae, Ga6A1	3.79
Ruminococcaceae, UCG_005	3.28	Rikenella	3.65
Eubacterium xylanophilum	3.27	Butyricicoccus	3.65
Anaeroplasma	3.24	Streptococcus	3.54
Candidatus Soleaferrea	3.23	Lachnospiraceae, A2	3.50
Ruminococcaceae, UCG_013	3.22	Lachnospiraceae, GCA_900066575	3.39
Clostridiales, Family XIII, AD3011	3.22	Eubacterium nodatum	3.38
Millionella	3.18	Ruminococcaceae, UCG_004	3.34
Ruminococcaceae, UCG_009	3.17	Turicibacter	3.27
Peptococcus	3.17	Prevotellaceae, UCG_001	3.20
Ruminococcaceae, UCG_014	3.12	Lachnospiraceae, UCG_006	3.15
Ruminococcaceae NK4A214	3.10	Blautia	3.11
Ruminococcaceae, UCG_010	3.10	Parasutterella	3.07
Ruminococcus 1	3.06	Muribaculum	3.03
Ureaplasma	3.02		
Pygmaiobacter	3.02		
Harryflintia	3.01		
Oxalobacter	3.01		
Fournierella	3.01		
Desulfovibrio	2.98		
Eubacterium ruminantium	2.91		

Table S35 (continued): More abundant bacterial genera within the cecum of each host species based on LEfSe analysis between *M. rufocanus* and *A. argenteus*.

Colon							
A. speciosus - A. argenteus				***A. speciosus - M. rufocanus***			
A. speciosus		***A. argenteus***		***A. speciosus***		***M. rufocanus***	
**Genus**	**LDA**	**Genus**	**LDA**	**Genus**	**LDA**	**Genus**	**LDA**
Tyzzerella 3	4.06	Campylobacter	4.42	Helicobacter	4.60	Treponema 2	4.00
Mucispirillum	3.86	Prevotellaceae, UCG_003	3.69	Rikenellaceae RC9	4.23	Ruminiclostridium	3.79
Eubacterium coprostanoligenes	3.44	Delftia	3.01	Bacteroides	4.19	Candidatus Saccharimonas	3.47
Ureaplasma	3.43	Brachyspira	2.76	Alistipes	4.10	Ruminococcaceae, UBA1819	3.38
Streptococcus	3.33	Eubacterium xylanophilum	2.59	Tyzzerella 3	4.06	Desulfovibrio	3.23
Ruminiclostridium 5	3.17	Oxalobacter	2.46	Mycoplasma	3.97	Eubacterium xylanophilum	3.20
Ruminococcaceae, UCG_014	3.13			Prevotellaceae, UCG_003	3.89	Ruminiclostridium 6	3.15
Prevotellaceae, UCG_001	2.83			Prevotellaceae Ga6A1	3.86	Millionella	3.07
Ruminococcaceae, UCG_010	2.67			Streptococcus	3.69	Ruminococcaceae NK4A214	3.04
Lachnospiraceae, UCG_006	2.44			Escherichia-Shigella	3.67	Ruminococcaceae, UCG_010	3.03
Ruminococcus 1	2.36			Oscillibacter	3.59	Ruminococcaceae, UCG_009	2.84
Enterorhabdus	2.30			Prevotella 9	3.43	Anaerostipes	2.79
				Leptotrichia	3.19	Clostridiales, Family XIII, AD3011	2.74
				Muribaculum	3.15	Ruminococcus 1	2.73
				Ruminiclostridium 5	3.07	Anaerovorax	2.71
				Prevotellaceae, UCG_001	3.06	Anaeroplasma	2.70
				Lachnospiraceae, GCA_900066575	2.96	Peptococcus	2.69
				Rikenella	2.88	Ruminococcaceae, UCG_005	2.60
				Gemella	2.85	Fournierella	2.57
				Eubacterium brachy	2.80	Pygmaiobacter	2.53
				Tyzzerella	2.78	Ruminococcaceae V9D2013	2.50
				Corynebacterium 1	2.61	Eubacterium ruminantium	2.48
				Lachnospiraceae, UCG_006	2.60	Quinella	2.48
				Parabacteroides	2.57	Oxalobacter	2.46
				Parasutterella	2.45	Lachnospiraceae NK4B4	2.42
				Turicibacter	2.45	Harryflintia	2.38
				Lachnoclostridium	2.40	Ruminococcaceae, GCA_900066225	2.36
				Blautia	2.40	Candidatus Soleaferrea	2.36
				Eubacterium nodatum	2.34	Spirochaetaceae, GWE2_31_10	2.35
				Butyricicoccus	2.12	Brachyspira	2.34
				Enterorhabdus	2.10	Erysipelotrichaceae, UCG_004	2.24
				Cerasicoccus	2.08		

Table S36: More abundant bacterial genera within the colon of each host species based on LEfSe analysis between *A. speciosus* and either *A. argenteus* or *M. rufocanus*. LDA scores shown.

Colon			
M. rufocanus - A. argenteus			
M. rufocanus		***A. argenteus***	
**Genus**	**LDA**	**Genus**	**LDA**
Ruminiclostridium	3.75	Mycoplasma	4.49
Candidatus Saccharimonas	3.49	Campylobacter	4.42
Ruminiclostridium 6	3.48	Alistipes	4.17
Ruminococcaceae, UBA1819	3.35	Bacteroides	4.02
Desulfovibrio	3.28	Rikenellaceae RC9	4.01
Eubacterium xylanophilum	3.21	Prevotella 9	3.90
Eubacterium coprostanoligenes	3.20	Prevotellaceae, UCG_003	3.86
Ruminococcaceae, UCG_010	3.17	Streptococcus	3.63
Ruminococcaceae, UCG_014	3.12	Prevotellaceae, Ga6A1	3.59
Mucispirillum	3.11	Rikenella	3.58
Ruminococcaceae NK4A214	3.08	Leptotrichia	3.40
Millionella	3.07	Eubacterium nodatum	3.09
Anaeroplasma	3.00	Muribaculum	2.94
Ruminococcaceae V9D2013	2.94	Prevotellaceae, UCG_001	2.84
Ruminococcus 1	2.94	Parasutterella	2.83
Ureaplasma	2.92	Lachnospiraceae, GCA_900066575	2.81
Ruminococcaceae, UCG_009	2.91	Turicibacter	2.79
Peptococcus	2.80	Lachnospiraceae, UCG_006	2.65
Pygmaiobacter	2.79		
Ruminiclostridium 5	2.66		
Fournierella	2.63		
Ruminococcaceae, UCG_005	2.57		
Candidatus Soleaferrea	2.50		

Table S36 (continued): More abundant bacterial genera within the colon of each host species based on LEfSe analysis between *M. rufocanus* and *A. argenteus*.

Rectum							
A. speciosus - A. argenteus				***A. speciosus - M. rufocanus***			
A. speciosus		***A. argenteus***		***A. speciosus***		***M. rufocanus***	
**Genus**	**LDA**	**Genus**	**LDA**	**Genus**	**LDA**	**Genus**	**LDA**
Helicobacter	4.18	Campylobacter	4.35	Bacteroides	4.41	Peptococcus	3.02
Mucispirillum	3.79	Rikenella	3.74	Helicobacter	4.26	Treponema 2	4.17
Streptococcus	3.67	Brachyspira	3.44	Alistipes	4.25	Bacteroidales, F082	4.12
Tyzzerella 3	3.57			Rikenellaceae RC9	4.17	Ruminococcus 1	3.88
Ureaplasma	3.28			Escherichia-Shigella	4.09	Eubacterium xylanophilum	3.75
Ruminococcaceae, UCG_002	2.87			Prevotellaceae Ga6A1	4.07	Candidatus Saccharimonas	3.70
Bilophila	2.74			Prevotellaceae, UCG_003	4.01	Ruminiclostridium	3.66
Enterorhabdus	2.52			Streptococcus	4.00	Ruminiclostridium 6	3.43
				Mycoplasma	3.92	Anaerofilum	3.39
				Mucispirillum	3.77	Desulfovibrio	3.25
				Oscillibacter	3.63	Acetatifactor	3.25
				Tyzzerella 3	3.56	Ruminococcaceae, UCG_010	3.19
				Prevotellaceae, UCG_001	3.43	Millionella	3.02
				Rikenella	3.41	Ruminococcaceae NK4A214	2.99
				Muribaculum	3.31	Pygmaiobacter	2.91
				Parasutterella	2.82	Anaeroplasma	2.90
				Eubacterium nodatum	2.73	Ruminococcaceae, UCG_005	2.87
				Lachnospiraceae, UCG_006	2.67	Fournierella	2.87
				Ruminococcaceae, UCG_004	2.63	Erysipelotrichaceae, UCG_004	2.86
				Parabacteroides	2.63	Ruminococcaceae, UCG_009	2.79
				Turicibacter	2.63	Ruminococcaceae V9D2013	2.78
				Butyricicoccus	2.42	Anaerostipes	2.75
						Lachnospiraceae FCS020	2.72
						Clostridiales, Family XIII, AD3011	2.64
						Eubacterium ruminantium	2.60
						Quinella	2.59
						Ruminococcaceae, GCA_900066225	2.56
						Lachnospiraceae NK4B4	2.52
						Candidatus Soleaferrea	2.48
						Oxalobacter	2.41
						Brachyspira	2.39
						Harryflintia	2.32

Table S37: More abundant bacterial genera within the rectum of each host species based on LEfSe analysis between *A. speciosus* and either *A. argenteus* or *M. rufocanus*. LDA scores shown.

Rectum			
M. rufocanus - A. argenteus			
M. rufocanus		***A. argenteus***	
**Genus**	**LDA**	**Genus**	**LDA**
Ruminococcus 1	3.88	Alistipes	4.42
Ruminiclostridium	3.82	Mycoplasma	4.38
Eubacterium xylanophilum	3.72	Rikenellaceae RC9	4.37
Candidatus Saccharimonas	3.71	Campylobacter	4.36
Ruminococcaceae, UBA1819	3.64	Bacteroides	4.35
Ruminiclostridium 6	3.34	Prevotellaceae, UCG_003	4.08
Desulfovibrio	3.28	Escherichia-Shigella	3.97
Acetatifactor	3.27	Prevotellaceae Ga6A1	3.91
Ruminococcaceae, UCG_010	3.25	Rikenella	3.90
Ruminococcaceae NK4A214	3.06	Streptococcus	3.73
Millionella	3.03	Eubacterium nodatum	3.33
Peptococcus	3.03	Prevotellaceae, UCG_001	3.10
Ruminococcaceae, UCG_014	3.03	Muribaculum	3.03
Anaeroplasma	3.01	Parasutterella	2.71
Pygmaiobacter	2.92	Lachnospiraceae, UCG_006	2.69
Fournierella	2.91	Turicibacter	2.49
Ruminococcaceae, UCG_009	2.89	Butyricicoccus	2.42
Ruminococcaceae, UCG_005	2.88		
Ruminococcaceae V9D2013	2.87		
Candidatus Soleaferrea	2.66		
Ureaplasma	2.64		
Clostridiales, Family XIII, AD3011	2.48		

Table S37 (continued): More abundant bacterial genera within the rectum of each host species based on LEfSe analysis between *M. rufocanus* and *A. argenteus*.
